# Decreasing *Bacillus thuringiensis israelensis* sensitivity of *Chironomus riparius* larvae with age indicates potential environmental risk for mosquito control

**DOI:** 10.1038/s41598-017-14019-2

**Published:** 2017-10-19

**Authors:** Anna Kästel, Stefanie Allgeier, Carsten A. Brühl

**Affiliations:** Institute for Environmental Sciences, University of Koblenz-Landau, Landau, Germany, Fortstrasse 7, 76829 Landau, Germany

## Abstract

Mosquito control based on the use of *Bacillus thuringiensis israelensis* (Bti) is regarded as an environmental friendly method. However, Bti also affects non-target chironomid midges that are recognized as a central resource in wetland food webs. To evaluate the risk for different larval stages of *Chironomus riparius* we performed a test series of daily acute toxicity laboratory tests following OECD guideline 235 over the entire aquatic life cycle of 28 days. Our study is the first approach that performs an OECD approved test design with Bti and *C. riparius* as a standard organism in ecotoxicological testing. First-instar larvae of *Chironomus riparius* show an increased sensitivity towards Bti which is two orders of magnitude higher than for fourth instar larvae. Most EC50 values described in the literature are based on acute toxicity tests using third and fourth instar larvae. The risk for chironomids is underestimated when applying the criteria of the biocide regulation EU 528/2012 to our data and therefore the existing assessment approval is not protective. Possible impacts of Bti induced changes in chironomid abundances and community composition may additionally affect organisms at higher trophic levels, especially in spring when chironomid midges represent a key food source for reproducing vertebrates.

## Introduction

*Bacillus thuringiensis* var. *israelensis* (Bti) formulations are commonly used agents for mosquito and black fly control worldwide^1,2^. More than 200 tons of Bti were applied annually in global mosquito control programs in the 1990s^3^. Bti is considered as the most environmental friendly alternative to chemical pesticides due to a high specifity to mosquito larvae and minimal effects to non-target organisms in closely related dipterans^4^. Within the group of Diptera the non-biting midges (Chironomidae) are the most Bti sensitive family^2^. In temperate regions chironomids are regarded as non-target organisms in mosquito control while in tropical countries they are also recognized as pests (and therefore target organisms) in rice culture^5^. In this case Bti is used as control agent for chironomids with maximum density reductions between 65% and 88% in experimental ponds^6,7^.

In most European mosquito control programs Bti products are usually applied over large areas by helicopter using a sling-bucket system while small wetlands are treated by hand^8^. In the Upper Rhine Valley (Germany) two different Bti formulations are used for mosquito control along 350 km of river: Vectobac® WG and Vectobac® 12 AS applied up to 12 times/season^9^. The nominal field rate depends on the occurring larval instars of the mosquito larvae, their density and flood water levels and is fixed at 1440 or doubled at 2880 ITU (International Toxic Units)/L^3,8,10^. Bti kills mosquito larvae by crystal and cytolitic-proteins that are built-up during sporulation of the bacteria^11^. Mosquito larvae consume these proteins which are activated in the alkaline milieu of the midgut subsequently. After activation they form pores in the epithelium leading to disruption of the midgut cells and finally to death of the larvae within a few hours^11,12^. The same mode of action takes place in the midgut of chironomids^13^.

Chironomids are the most abundant group among aquatic macroinvertebrates in aquatic habtitats^14–16^. The life cycle of chironomids comprises four larval instars, a pupal life stage and the flying midge as imago^14,15^. Their ubiquity, species richness, high abundances and high ecological diversity in all kinds of lentic and lotic habitats make them a central food resource in wetland food webs^14^. Adult chironomids form huge swarms and can dominate insect emergence in wetlands with over 90% of the emerging individuals^17^. Additional to their availability and high biomass ranging between 1.0 and 100 g dry weight per year and square meter^14^ chironomid larvae have a high protein content and digestibility^18^. All in all, chironomids are not only a frequent but also a valuable food resource for various insects and crustaceans as well as amphibians, birds, fish and mammals at higher trophic levels^14^.

Chironomid larvae are routinely tested as standard organism representing aquatic insects in the environmental risk assessment for pesticides^19,20^. Acute toxicity is measured with first instar larvae as value for the effective concentration where 50% of the individuals are immobile (EC50)^19^. Since mortality is difficult to assess in first-instar larvae immobility is used as alternative to mortality. Therefore EC50 and LC50 values (lethal concentration where 50% of the individuals are dead) are equivalent. Acute laboratory tests are conducted without sediment to represent a worst case scenario. The first larval instar of chironomids is free-swimming and hence not affected by the absence of sediment^20^. Furthermore, first instar chironomids showed a higher sensitivity to certain stressors such as heavy metals and chemicals^21,22^. The sensitivity regarding the EC50 values between first and fourth larval stages of *C. riparius* could differ by e.g. a factor up to 950 for Cadmium^23^. Although Bti products are applied directly to water bodies no EC50 values for first instar chironomids could be found in the literature and documents for Bti product registrations^24^.

In Europe Bti (Serotype H-14, strain AM65-52) is regulated as biocide under the guideline 528/2012. The guideline pursues the protection of non-target organisms, the environment, biodiversity and ecosystems^25^. The crucial instrument for granting authorization is the PEC (Predicted environmental concentration)/PNEC (Predicted no effect concentration) ratio which should not exceed 1 to assure previously mentioned protection goals. Since 2011, Bti is approved under the former biocide directive 98/8/EC. For the latest assessment report^26^ no ecotoxicological values for Chironomidae were available and could be included in the risk assessment.

Several toxicity studies produced EC50 values to pestiferous chironomid larvae using effective concentrations for different products and study designs. However, data are only available for third and fourth larval instars^22,23,27^. In contrast to these efficiency studies we tested larvae of *C. riparius* as a non-target organism. We followed the standardized study design according to OECD (Organization for Economic Co-operation and Development) Guideline 235 to obtain comparable 48 h EC50 values for different larval stages^19^. These tests were conducted daily over the entire aquatic life cycle of 28 days in order to test how sensitivity changes during larval development. Mean EC50 values for every larval stage were calculated and compared to reviewed literature values and field application rates for mosquito control. Furthermore we compared and evaluated the different EC50 values of previous studies that addressed Bti sensitivity of chironomids. PEC/PNEC ratios for the different species and larval instars were calculated to simulate a risk assessment under the guideline EU 528/2012^25^.

## Results

**Figure 1:**
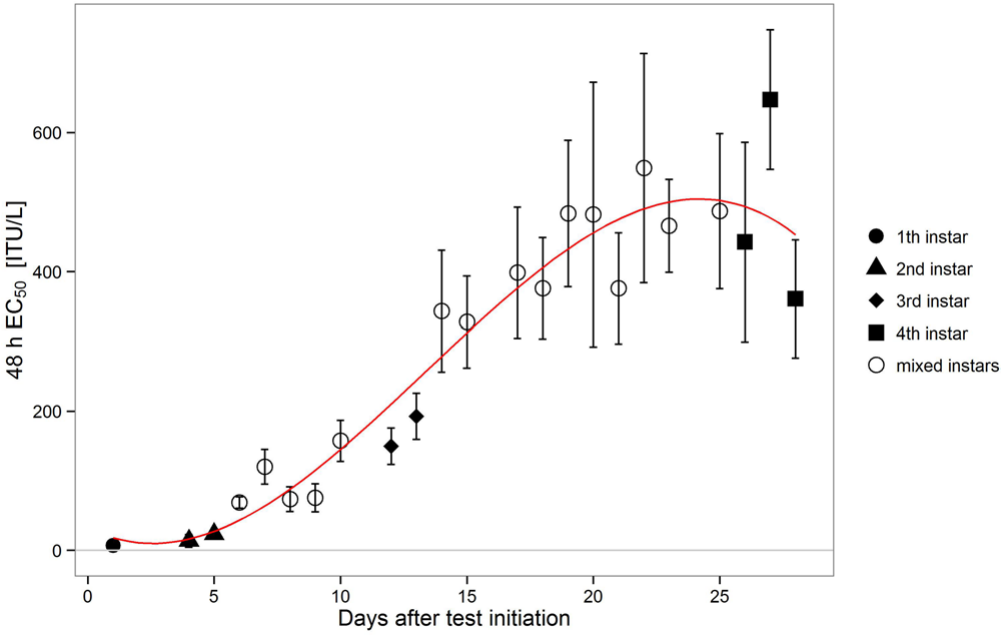
EC50 values with 95% Confidence Intervals (CI) on each test day during the 28 day test period. Days with more than 90% of the individuals attributed to a specific larval stage were assigned to first until fourth instars (filled symbols). The EC50 values where this criterion was not met are marked as mixed instars (unfilled symbol). Red line: curve fit of the EC50 values f(x) = 37.2 − 20.1x + 4.1x^2^ − 0.1x^3^, adjusted R^2^ = 0.92, p = 1.165e-10. For each day 150 larvae were used.

During its larval development *Chironomus riparius* showed a broad spectrum of sensitivity to Bti with EC50 values ranging from 6.9 ITU/L up to 607.8 ITU/L (Fig. 1). The first and most sensitive larval instar was 100-fold more sensitive than the fourth larval instar. Within this range the decrease in sensitivity of developing larval stages could be described with a sigmoid curve fit (f(x) = 37.2 − 20.1x + 4.1x^2^ − 0.1x^3^, adjusted R^2^ = 0.92) suggesting an overall decrease in sensitivity for older larvae (except for the last days, when pupation occurred, and individuals stop feeding and are therefore also not exposed to Bti).

In four of 28 tests the control immobilisation exceeded 15% or did not achieve 100% immobilization in the highest concentration which is why these studies were excluded from the analysis and do not appear in Fig. 1 (see Supplemental Information, Table S1). Days with less than 90% individuals of the same larval instar stage in the corresponding test are declared as ‘mixed instars’ (further details can be found in Table S1, Supplemental Information). Out of 28 tests 8 were identified as mixed instars even though all larvae were from one age cohort (within 24 h).

For each of the four larval stages mean EC50 values were calculated and compared to each other. Mean EC50 values increase with successive larval stage (Table 1). All mean EC50 values are statistically significantly different from each other with p-values below 0.003 (Supplemental Information, Table S10) and separated by factors between 2.3 and 10.5. The highest increase is between second and third larval instar. First and second instar larvae show a high sensitivity to Bti compared to older larval stages.

**Table 1:** Mean EC50 values and 95% confidence intervals. All mean EC50 are statistically significant different from each other.

Larval instar	Mean EC50 (ITU/L)	95% Confidence Interval (ITU/L)	Included test days
First	6.9	3.8–10.0	2
Second	16	13.6–18.4	4, 5
Third	168.7	147.9–189.4	12, 13
Fourth	485	416.6–553.3	26, 27, 28

Included test days (see main text) are provided.

**Figure 2:**
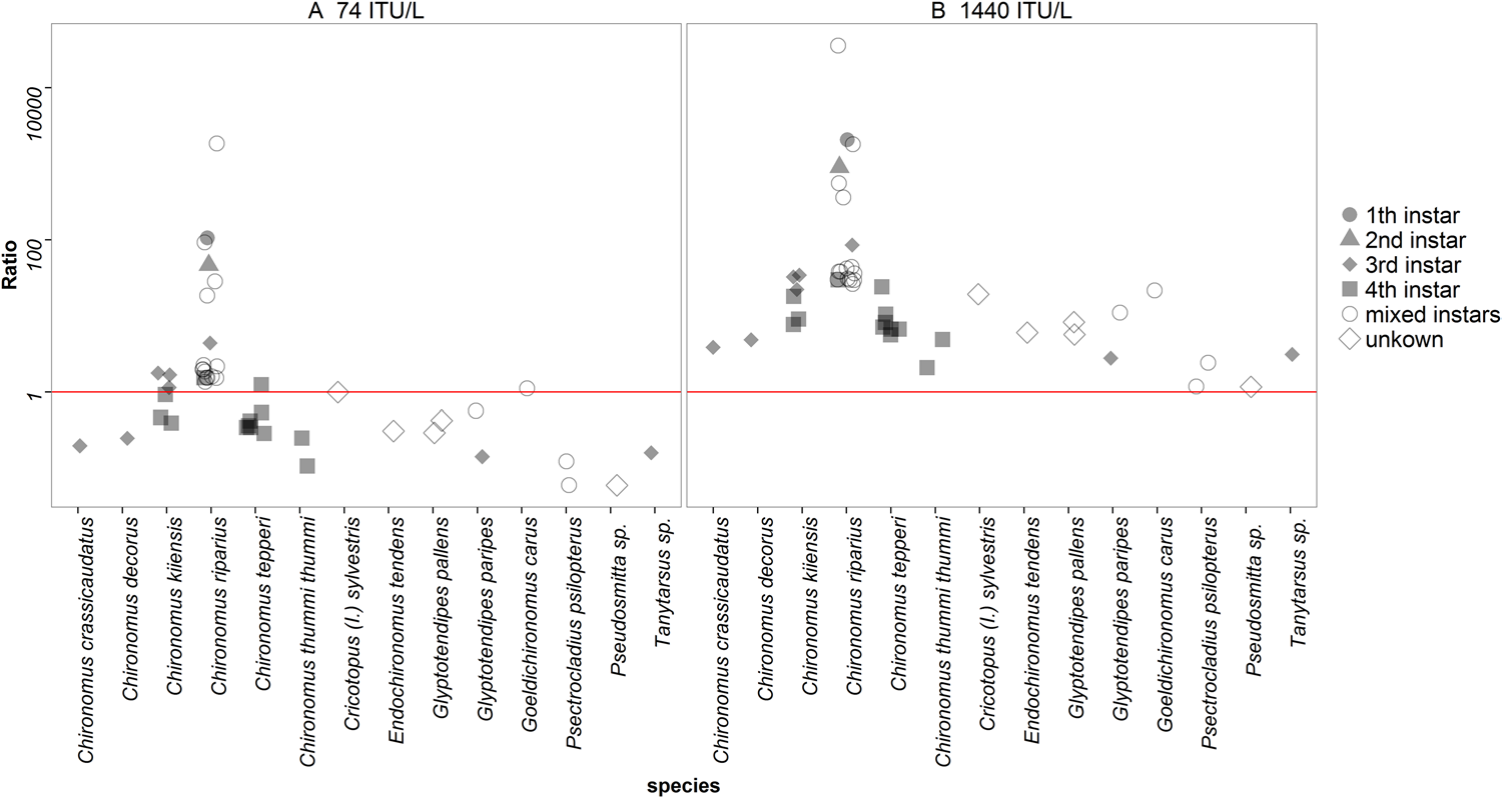
The calculated ratio of PEC/PNEC is shown. The ratio was calculated with a PEC of 74 ITU/L (**A**) respectively 1440 ITU/L which is the field rate (**B**). The red line marks the trigger value of the biocide guideline (PEC/PNEC = 1).

The PEC/PNEC ratio for the biocide risk assessment was calculated using two different PEC values. One was obtained from the risk assessment report of Bti Serotype H-14, strain AM65-52 (74 ITU/L)^26^; the other (1440 ITU/L) is the actual exposure concentration in surface water in mosquito control areas in the Upper Rhine Valley^10^. PNEC values are based on EC50 values of the different species obtained from literature (Supplemetal Information, Table S3). For the lower exposure value four species exceed the trigger value 1 of the exposure/effect ratio (Fig. 2A). However at actual mosquito control rates all chironomid species are at risk since the PEC/PNEC ratio exceeds 1 in all cases (Fig. 2B). The most relevant value for the risk assessment is the EC50 of first instar larvae of *C. riparius*. Here the exposure/effect ratio is 105 (PEC = 74 ITU/L) or 2057 respectively (PEC = 1440 ITU/L). Both exceed the trigger value of 1 by several orders of magnitudes indicating an underestimated risk (Fig. 2).

## Discussion

Our study reveals a high sensitivity of first and second instar larvae of *C. riparius*, with EC50 values 209 times (in case of first instar larvae) and 90 times (in case of second instar larvae) below the lowest field application concentration used in mosquito control in the Upper Rhine Valley, Germany. The results of the consecutive test design of this study showed a decrease in sensitivity for a cohort of chironomid larval instars towards Bti exposure during their development. Bti sensitivity was significantly different for all larval instars of *C. riparius* in this study.

Earlier larval instars of chironomids are more susceptible to Bti than older instars. The factors separating the larval instars are not consistent within the different tested species. Reported factors of decrease differ for different instars and species from 4-fold^21^ to 174-fold^28^ but the trend, older larvae react less sensitive, is consistent^29^. The differences could be caused by the variance in test design regarding species and size of the larvae or larval densities. The size of the larvae matters, because - even in the same larval instar - EC50 values of Bti were reported statistically significant different^6^. Also the different Bti formulations could affect chironomid sensitivity^5^. Inert ingredients comprise a major part of the formulations and are suspected to change the settling rate of the Bti product or the feeding behavior of the larvae^5^.

The actual assessment report on Bti for its registration in the EU did not include the most sensitive non-target organism – Chironomidae - but referred to the crustacean *Daphnia magna* instead^26^. Chironomids would be more suitable due to their close relationship to the target organism mosquito. They live in the targeted environment and the uptake and the mode of action of Bti is similar. Additionally midges are also recognized as central food resource in wetlands^30–33^. Following the Guideline 528/2012 the PEC/PNEC ratios exceed the trigger value for all reviewed chironomid species^25^. Most studies tested less sensitive instars, so the presumed safety of Bti for non-target Chironomidae is not given. In case of the first instar larvae of *C. riparius* the PEC/PNEC ratio is 2057 which is more than 2000 times higher than acceptable. Based on the violation of the PEC/PNEC ratio Bti and its formulated products need a reevaluation of the existing approval. Potential environmental harm is indicated by including our sensible and sensitive endpoint in the risk assessment.

The acute toxicity test is a worst case scenario for chironomid larvae. In the field the sensitivity of chironomids to Bti could be lower due to the presence of sediment, sunlight and other abiotic and biotic factors^34–36^. However, the exposure rates of 74 ITU/L and 1440 ITU/L used in our risk calculation are situated at the lower end of the existing range; field rates in Europe generally vary between 1,440 ITU/L and 3,198 ITU/L^10,31,37–39^. Field studies monitoring mosquito control in wetlands sometimes detected reductions of chironomid populations^37,40–42^, others did not find any effects^38,43–45^. Community composition of chironomid species and larval instars in mosquito breeding sites with Bti application is often unknown. Field populations consist of a mixture of different larval instars^46^ which lead to different sensitivity levels. Another possible explanation for the varying results in the field studies is the different species composition of the aquatic insect community which arises from different habitats like salt marshes, river floodplains and seasonal wetlands.

Chironomids represent non-target organisms in mosquito control scenarios^2,40^. Due to their ubiquitous occurrence and high numbers they are one of the most valuable food resources in temporary wetlands^14,47^. A decline of chironomids alongside with the removal of mosquito larvae leads to a reduction of available biomass for organisms at higher trophic levels and thus has implications for the entire food web^31,33,37,41^. Various predators feed directly and indirectly on chironomid larvae and imagines like dragonflies, spiders, amphibians and their larvae, fish, birds and bats^14,31,37,48–51^. Some studies exist on direct and indirect effects on higher trophic levels after Bti application^37,52^ and only a thorough evaluation of their study designs and data analysis together with further coordinated research can help to come to a valid conclusion regarding potential food web deterioration due to Bti mosquito control. In Germany, in contrast to other countries as Sweden, USA or France no long-term environmental monitoring with control sites was established to allow a solid analysis^31,38,41,43^.

The results from this laboratory study indicate that the risk for chironomids in the course of Bti-based mosquito control is underestimated. This could lead to disruptions on higher trophic levels within the wetland food web. As an environmental friendly alternative to other insecticides^3^, in Germany Bti is also applied multiple times per season in nature conservation areas of European value with specific protected target species^10,31,40^. Currently the magnitude of Bti effects on wetland food webs is unknown and nature protection goals might be violated.

## Material and Methods

### Test organism

The test organism *Chironomus riparius* Meigen 1804 (obtained from BayerCropScience AG, Monheim 2013) was kept in permanent culture within a climate controlled chamber (Weiss Environmental Technology Inc., Germany) at 20 ± 1 °C with a 16:8 light/dark regime with 800–1000 lux light intensity. Animals were cultured in M4 Medium^19^ which was renewed once a week. The culture vessels with larvae were gently aerated and a layer of quartz sand (0.5 mm) was provided. Larvae were fed with ground fish food (TetraMin, Germany).

### Rearing the tested larvae

20 fertile egg ropes not older than 24 h were collected three days before test initiation and reared in separate culture vessel without any sediment but aeration. Ground fish food was added every two days. To reduce stress resulting from high density larvae were randomly separated into different vessels after five days. Medium was renewed whenever necessary but latest after three days. The larvae were reared in the climatic chamber mentioned above.

### Acute toxicity tests

The acute toxicity tests were conducted according to OECD guideline 235. Five larvae were exposed to five different test concentrations in 100 mL plastic beakers (Duny, Bramsche, Germany) filled with 50 mL test solution, prepared with M4 medium and Vectobac WDG. Each of the five treatment concentrations (Supplemental Information, Table S1) and the M4 medium control consisted of five replicates. Individuals that did not move after a gentle stream produced with a pipette were considered as moribund. The number of immobile individuals was recorded after 24 h and 48 h. Individuals of the 4^th^ instar larvae that started pupation during the test were excluded from the test^47^. Consequently the number of larvae per beaker deviated from five in the highest larval instar tests after 26 days. During the tests no food and aeration was provided. All tests ran in the climatic chamber as described previously. Oxygen content and pH was measured at the end of each test (after 48 h) and was always found in agreement with OECD Guideline 235.

### Test substance and concentrations

The test substance Vectobac WDG (Valent BioSciences Corporation, USA) has the toxic potency of 3000 International Toxic Units (ITU) per mg. The active ingredient is *Bacillus thuringiensis israelensis* (strain AM 65–52). VectoBac WDG was sterilized by gamma radiation according to the standard procedure of the German Mosquito Control Association (KABS e. V.). Thereafter the potency of Vectobac WDG is reduced to approximately 2400 ITU/mg^53^.

Due to larval development and decreasing sensitivity the test concentrations were adjusted during the test period of 28 days (further details in Supplemental Information, Tables S1 and S2). A solution with a certain amount of VectoBac WDG was prepared in M4 medium. The amount of Bti was weighed and a stock solution was prepared. The stock solution was diluted further using M4 medium until the desired test concentrations were achieved (details for preparation in Supplemental Information Table S2). To allow comparison with other studies the concentration is not given in mg VectoBac WDG/L but in ITU/L.

### Determination of larval stages

Head capsule measurements were conducted to determine the larval stage of *C. riparius* since the age in days or body length is not a sufficient method to determine the larval instar^54^. Each day 10 to 20 randomly selected larvae were taken out of the rearing vessel, preserved in 70% Ethanol and head capsule width (HCW) and head capsule length (HCL) were determined using a binocular microscope (Leica CME, Leica Microsystems, Germany) fitted with a calibrated eyepiece micrometer.

HCW and HCL of the selected individuals were analyzed with k-mean clustering (vegan package, R) and assigned to one of the four clusters corresponding to four larval instars. Percentages of the different larval instars were calculated daily (further details in Supplemental Information, Tables S1, S6 and Figure S7).

### Risk assessment for Biocides

The PEC/PNEC ratio was calculated following the Guideline EU 528/2012. PNEC is extrapolated from the EC50 value of the most sensitive organism, in this case first larvae of *C. riparius* and the assessment factor of 10. This leads to a PNEC of 0.69 (6.9 ITU/L: 10 = 0.69 ITU/L). The PNEC was calculated for all reported EC50 values. Differing test parameters of the nine evaluated studies were summarized (Supplemental Information, Table S3, Figure S6, Table S7). The PEC was derived from the assessment report of Italy and stated as 74 ITU/L, which is the lowest possible PEC in the report^26^. The resulting concentration after application to surface water in Germany is 1,440 ITU/L, and this was included as a realistic value in the analysis.

### Statistical analyses

All statistical analyses were carried out using the statistical software R (version 3.1.0)^55^. The significance level to detect differences was set to α = 0.05 for all tests. Dose-response models in the drc package^56^ were fitted to the data and the daily 48 h EC50 with 95% Confidence Interval was calculated with the best model. Model fit was assessed using Akaike’s information criterion.

Tests were considered valid if the control mortality did not exceed 15% as recommended in the OECD Guideline 235 for acute toxicity tests^19^. As mentioned above larvae for headcapsule measurements were randomly selected every day. The headcapsule measurements of these larvae allowed conclusions about the instar stage. The percentage of larvae in respective larval instar was calculated for every test day (Supplemental Information, Table S1). When more than 90% of the larvae were found to be in the same larval stage, the test on this day was assigned to this larval instar (Supplemental Information, Table S1). Test days which fulfilled the criterion of 90% larvae within the same instar, less than 15% control mortality and 100% mortality in the highest concentration were used for mean EC50 calculations. Data of every larval stage were fitted to a dose-response model (Supplemental Information Figure S5, Table S3). Mean EC50 values were analysed for statistically significant differences among the four larval instars using confidence interval overlap testing (Supplemental Information, Table S10)^57^. To extract the most influential parameters of the EC50 values obtained from the literature review a linear model was calculated with the “car” package^58^. Different linear models were tested with ANOVA for significant difference to get the most parsimonious model (Supplemental Information Figure S6, Table S7).

## Electronic supplementary material

Supplementary Information
