# Supplementary material for: Decreasing Bacillus thuringiensis israelensis sensitivity of Chironomus riparius larvae with age indicates potential environmental risk for mosquito control
Source: Sci Rep. 2017 Oct 19;7:13565. doi: 10.1038/s41598-017-14019-2 (PMC5648803; doi:10.1038/s41598-017-14019-2)
Supplement: Supplementary file 1 — Supplementary Information [file 41598_2017_14019_MOESM1_ESM.pdf]

Decreasing *Bacillus thuringiensis israelensis* sensitivity of *Chironomus riparius* larvae with age indicates potential environmental risk for mosquito control

Supplemental information

Author list:

Anna Kästel (kaestel@uni-landau.de)[A]

Stefanie Allgeier (allgeier@uni-landau.de)[A]

Carsten A. Brühl (bruehl@uni-landau.de)* [A]

[A] Institute for Environmental Sciences, University of Koblenz-Landau, Landau, Fortstrasse 7, 76829 Landau, Germany

* Corresponding author:

Carsten Brühl
Mail bruehl@uni-landau.de
Phone +49 (0)6341 280-31310

Institute for Environmental Sciences
University Koblenz-Landau
Fortstraße 7
D-76829 Landau
Germany

Table S1: Each test day is shown with the absolute and relative number of chironomid larvae in the different larval instars based on headcapsule measurements. The test concentration of Vectobac WDG and the spacing factor between concentrations are given. The range of the tested Vectobac WDG concentrations and their spacing factor are given. Control mortality for each test day was implemented in Abotts formula unless it exceeded 15% (according to OECD Guideline 235) the test days were excluded from further analysis by fullfilling the criterions the test day was assigned to one of the four larval instars.

Test	Instar (Number)				Instar (%)				Concentration range	Spacing factor	Control mortality	Assigned larval
day	1th	2nd	3rd	4th	1th	2nd	3rd	4th	Vectobac WDG			instar
1	12	-	-	-	100	0	0	0	3.2-125 ITU/L	2.5	0%	1
2	11	-	-	-	100	0	0	0	3.2-125 ITU/L	2.5	21%	x
3	5	6	-	-	45	55	0	0	3-243 ITU/L	3	20%	x
4	-	11	-	-	0	100	0	0	3-243 ITU/L	3	0%	2
5	-	11	1	-	0	92	8	0	15-240 ITU/L	2	8%	2
6	-	6	2	-	0	75	25	0	15-240 ITU/L	2	4%	x
7	-	8	5	-	0	62	38	0	25-400 ITU/L	2	4%	x
8	-	6	9	-	0	40	60	0	25-400 ITU/L	2	0%	x
9	-	10	8	-	0	56	44	0	30-480 ITU/L	2	4%	x
10	-	2	14	-	0	13	88	0	50-800 ITU/L	2	0%	x
11	-	-	11	1	0	0	92	8	50-800 ITU/L	2	4%	x
12	-	-	13	1	0	0	93	7	100-1600 ITU/L	2	0%	3
13	-	-	12	-	0	0	100	0	125-2000 ITU/L	2	0%	3
14	-	-	8	4	0	0	67	33	125-2000 ITU/L	2	4%	x
15	-	-	11	8	0	0	58	42	187.5-3000 ITU/L	2	0%	x
16	-	1	14	5	0	5	70	25	187.5-3000 ITU/L	2	16%	x
17	-	-	12	5	0	0	71	29	187.5-3000 ITU/L	2	12%	x
18	-	-	10	10	0	0	50	50	187.5-3000 ITU/L	2	4%	x
19	-	-	8	10	0	0	44	56	200-3200 ITU/L	2	4%	x
20	-	-	9	8	0	0	53	47	200-3200 ITU/L	2	12%	x
21	-	-	9	7	0	0	56	44	200-3200 ITU/L	2	12%	x
22	-	-	6	10	0	0	38	63	250-4000 ITU/L	2	4%	x
23	-	-	7	11	0	0	39	61	250-4000 ITU/L	2	8%	x
24	-	-	4	9	0	0	31	69	250-4000 ITU/L	2	16%	x
25	-	-	4	13	0	0	24	76	250-4000 ITU/L	2	8%	x
26	-	-	-	17	0	0	0	100	250-4000 ITU/L	2	4%	4
27	-	-	-	17	0	0	0	100	250-4000 ITU/L	2	4%	4
28	-	-	1	11	0	0	8	92	250-4000 ITU/L	2	0%	4
29	-	-	2	10	0	0	17	83	250-4000 ITU/L	2	12%	x
30	-	-	-	19	0	-	-	100	250-4000 ITU/L	2	8%	x

Table S2: Detailed test information on the preparation of stock solutions and dilution series are provided. The following pipets were used for respective amounts: 5 - 10 mL: Pipetman P10 (Gilson, France); 1-4.9 mL: Pipetman P5000 (Gilson, France); < 1 ml: Pipetman P1000 (Gilson, France). A Mettler AT261 Deltarange balance was used for weighing respective amounts of Vectobac WDG. Concentrations were prepared and measured in volumentric flasks. The volumes marked with asteriks were taken in 2 steps: 10 mL pipeting and 3.5 mL pipeting.

Date (2015)	Test Day	stock 1		stock 2		concentration 5		concentration 4		concentration 3		concentration 2		concentration 1	
		amount Bti (mg)	Volume (mL)	amount stock1 (mL)	Volume (mL)	stock (mL)	Volume (mL)	stock (mL)	Volume (mL)	stock (mL)	Volume (mL)	stock (mL)	Volume (mL)	stock (mL)	Volume (mL)
16.02.	1	16.7	1000	5	500	250	1000	25	250	10	250	4	250	1.6	250
17.02.	2	16.7	1000	5	500	250	1000	25	250	5	250	4	250	1.6	250
18.02.	3	20.0	2000	25	500	40.5	250	13.5*	250	4.5	250	1.5	250	0.5	250
19.02.	4	20.0	2000	25	500	40.5	250	13.5*	250	4.5	250	1.5	250	0.5	250
20.02.	5	16.7	1000	10	500	60	250	30	250	15	250	7.5	250	3.75	250
21.02.	6	16.68	1000	10	500	60	250	30	120	15	250	7.5	250	3.75	250
22.02.	7	16.7	1000	25	250	20	250	10	250	5	25	2.5	250	1.25	250
23.02.	8	16.7	1000	25	250	20	250	10	250	5	250	2.5	250	1.25	250
24.02.	9	24.9	1000	10	100	16	250	8	250	4	250	2	250	1	250
25.02.	10	41.7	1000	10	100	16	250	8	250	4	250	2	250	1	250
26.02.	11	41.7	1000	10	100	16	250	8	250	4	250	2	250	1	250
27.02.	12	166.7	2000	10	100	16	250	8	250	4	250	2	250	1	250
28.02.	13	52.1	500	10	100	16	250	8	250	4	250	2	250	1	250
01.03.	14	52.1	500	10	100	16	250	8	250	4	250	2	250	1	250
02.03.	15	62.5	2000	-	-	8	250	4	250	2	250	1	250	0.5	250
03.03.	16	62.5	2000	-	-	8	250	4	250	2	250	1	250	0.5	250
04.03.	17	62.5	2000	-	-	8	250	4	250	2	250	1	250	0.5	250
05.03.	18	62.5	2000	-	-	8	250	4	250	2	250	1	250	0.5	250
06.03.	19	16.7	1000	-	-	16	250	8	250	4	250	2	250	1	250
07.03.	20	16.7	1000	-	-	16	250	8	250	4	250	2	250	1	250
08.03.	21	16.7	1000	-	-	16	250	8	250	4	250	2	250	1	250
09.03.	22	41.7	1000	-	-	8	250	4	250	2	250	1	250	0.5	250
10.03.	23	41.7	1000	-	-	8	250	4	250	2	250	1	250	0.5	250
11.03.	24	41.7	1000	-	-	8	250	4	250	2	250	1	250	0.5	250
12.03.	25	41.7	1000	-	-	8	250	4	250	2	250	1	250	0.5	250
13.03.	26	41.7	1000	-	-	8	250	4	250	2	250	1	250	0.5	250
14.03.	27	41.7	1000	-	-	8	250	4	250	2	250	1	250	0.5	250
15.03.	28	41.7	1000	-	-	8	250	4	250	2	250	1	250	0.5	250

Table S3: The following table summarizes information on toxicity tests with *Bacillus thuringiensis israelensis* on different chironomid species obtained from the literature review. The EC50 values in the literature were stated in different units but were converted to ITU/L. Parameters known to influence the EC50 value for chironomids are provided.

**Species**	**Larval instar**	**EC50 (ITU/L)**	**Test duration (h)**	**95 % Confidence Interval (ITU/L)**	**Product**	**Author**	**Larvae per replicate**	**Substrate**
Chironomus crassicaudatus	3	3750	48	NA	IPS-78 (WP)	Ali et al 1981	20	sand
Chironomus decorus	3	2980	48	NA	IPS-78 (WP)	Ali et al 1981	20	sand
Glyptotendipes paripes	3	5210	48	NA	IPS-78 (WP)	Ali et al 1981	20	sand
Tanytarsus sp.	3	4630	48	NA	IPS-78 (WP)	Ali et al 1981	20	sand
Glyptotendipes paripes	mix of 3+4	1307	48	948 - 1673	Technical powder	Ali et al 2008	20	sand
Goeldichironomud carus	mix of 3+4	665	48	441 - 904	Technical powder	Ali et al 2008	20	sand
Cricotopus (I.) sylvestris	not stated	748	48	NA	Bactimos PP	Fillinger 1998	15	none
Endochironomus tendens	not stated	2398	48	1111 - 2838	Bactimos PP	Fillinger 1998	15	none
Glyptotendipes pallens	not stated	1749	48	NA	Bactimos PP	Fillinger 1998	12	none
Glyptotendipes pallens	not stated	2541	48	550 - 3949	Bactimos PP	Fillinger 1998	17	none
Pseudosmittia sp.	not stated	12419		5775 - 19635000	Bactimos PP	Fillinger 1998	25	none
Chironomus thummi thummi	4	2970	24	2790 - 3150	Bactimos powder BRB 0032	Yiallouros et al 1999	25	none
Chironomus thummi thummi	4	6930	24	6300 - 7560	Bactimos powder BRB 0032	Yiallouros et al 1999	25	none
Psectrocladius psilopterus	mix of 3+4	12285	24	6510 - 18060	Bactimos powder BIB 0036	Yiallouros et al 1999	25	none
Psectrocladius psilopterus	mix of 3+4	5985	24	5355 - 6615	Bactimos powder BIB 0036	Yiallouros et al 1999	25	none
Chironomus kiiensis	4	804	24	360 - 1260	oil miscible supension	Cao et al 2012	30	none
Chironomus kiiensis	4	1884	24	948 - 3072	wettable powder	Cao et al 2012	30	none
Chironomus kiiensis	4	1600	24	800 - 2650	technical material	Cao et al 2012	30	none
Chironomus kiiensis	3	420	24	180 - 696	oil miscible supension	Cao et al 2012	30	none
Chironomus kiiensis	3	444	24	168 - 816	wettable powder	Cao et al 2012	30	none
Chironomus kiiensis	3	650	24	150 - 1350	technical material	Cao et al 2012	30	none
Chironomus tepperi	4	1380	48	1260 - 1530	VectoBac WDG	Stevens et al 2004	10	sand
Chironomus tepperi	4	600	48	420 - 870	VectoBac WDG	Stevens et al 2004	10	sand
Chironomus tepperi	4	1770	48	1680 - 1890	VectoBac WDG	Stevens et al 2005	10	sand
Chironomus tepperi	4	2040	48	1668 - 2496	Aquabac SC	Stevens et al 2005	10	sand
Chironomus tepperi	4	2580	48	2268 - 2928	Teknar SC	Stevens et al 2005	10	sand
Chironomus riparius	1	7	48	4 - 10	VectoBac WDG	Kästel	5	none
Chironomus riparius	2	16	48	14 - 18	VectoBac WDG	Kästel	5	none
Chironomus riparius	3	169	48	148 - 189	VectoBac WDG	Kästel	5	none
Chironomus riparius	4	485	48	417 - 553	VectoBac WDG	Kästel	5	none
Chironomus riparius	mix of 3+4	0.4	48	0.004 - 2	Vectobac G	Chabonneau et al 1994	10	sediment
Chironomus riparius	mix of 3+4	8	48	4 - 22	Vectobac G	Chabonneau et al 1994	10	sediment
Chironomus riparius	mix of 3+4	40	48	16 - 102	Vectobac G	Chabonneau et al 1994	10	soil
Chironomus riparius	mix of 3+4	26	48	2 - 200	Vectobac G	Chabonneau et al 1994	10	soil
Chironomus tepperi	4	2160	48	1860 - 2520	Bactimos	Treverrow 1985	20	NA

Table S4: Detailed information on EC50 calculations of the selected model on each test day. 95% Confidence Interval (CI), lack of fit and Akaikes information criterion were calculated with the package “drc” in R.

Test day	EC50	lower CI	upper CI	Model		Akaikes Information Criterion	Lack of fit
Day 1	6.87	3.77	9.98	log-logistic dose-response model with two parameters	LL.2	-10.14	0.80
Day 2	25.70	17.50	33.90	two-parameter Weibull function	W1.2	-2.14	0.92
Day 3	14.93	9.00	20.85	two-parameter Weibull function	W1.2	-6.25	0.83
Day 4	13.64	4.63	22.65	log-normal dose-response model with four parameter	LN.4	-19.37	0.00
Day 5	23.33	18.95	27.71	two-parameter Weibull function	W2.2	-26.90	1.00
Day 6	68.73	60.64	76.83	two-parameter Weibull function	W1.2	-37.79	0.97
Day 7	120.00	95.00	144.99	two-parameter Weibull function	W2.2	-24.73	0.52
Day 8	73.67	56.03	91.31	log-normal dose-response model with two parameter	LN.2	-13.44	0.82
Day 9	75.58	55.36	95.80	log-normal dose-response model with two parameter	LN.2	-6.13	0.36
Day 10	157.50	127.88	187.12	two-parameter Weibull function	W1.2	-17.74	0.74
Day 11	371.34	319.22	423.47	two-parameter Weibull function	W1.2	-45.48	0.52
Day 12	149.72	123.60	175.85	two-parameter Weibull function	W1.2	-23.54	0.87
Day 13	192.60	159.54	225.67	two-parameter Weibull function	W1.2	-31.17	0.96
Day 14	343.33	256.08	430.59	two-parameter Weibull function	W2.2	-19.76	0.01
Day 15	328.04	262.00	394.07	log-normal dose-response model with two parameter	LN.2	-16.95	0.97
Day 16	434.11	346.52	521.70	two-parameter Weibull function	W1.2	-15.02	0.33
Day 17	398.77	304.57	492.97	two-parameter Weibull function	W2.2	-29.04	0.48
Day 18	376.13	303.42	448.83	two-parameter Weibull function	W1.2	-27.12	0.88
Day 19	483.85	378.71	588.99	log-logistic dose-response model with two parameters	LL.2	-22.55	0.85
Day 20	482.16	291.90	672.41	two-parameter Weibull function	W2.2	-2.15	0.67
Day 21	375.99	296.31	455.67	two-parameter Weibull function	W1.2	-18.09	1.00
Day 22	549.06	384.40	713.73	two-parameter Weibull function	W2.2	-14.28	0.50
Day 23	465.76	398.96	532.55	two-parameter Weibull function	W2.2	-37.50	0.49
Day 24	410.31	327.51	493.12	two-parameter Weibull function	W2.2	-10.95	0.88
Day 25	487.02	375.72	598.31	log-normal dose-response model with two parameter	LN.2	-13.13	1.00
Day 26	442.63	299.22	586.05	two-parameter Weibull function	W2.2	-4.72	0.95
Day 27	647.26	547.00	747.52	two-parameter Weibull function	W2.2	-32.71	0.94
Day 28	361.07	276.33	445.81	log-normal dose-response model with two parameter	LN.2	-18.02	0.99

Figure S5: 48h EC50 value (red dot) with its 95% CI on each test day. All replicates of the test are included in the figures below (transformed with Abbotts formula to adjust for control mortality). Tests with control mortality exceeding 15 % (Day 2,3,16 and 24) were excluded from further analysis but are presented here for completeness. The results for study Day 11 was excluded from further analysis because the mortality in the highest concentration was too low (72%) to produce a reliable EC50 value.

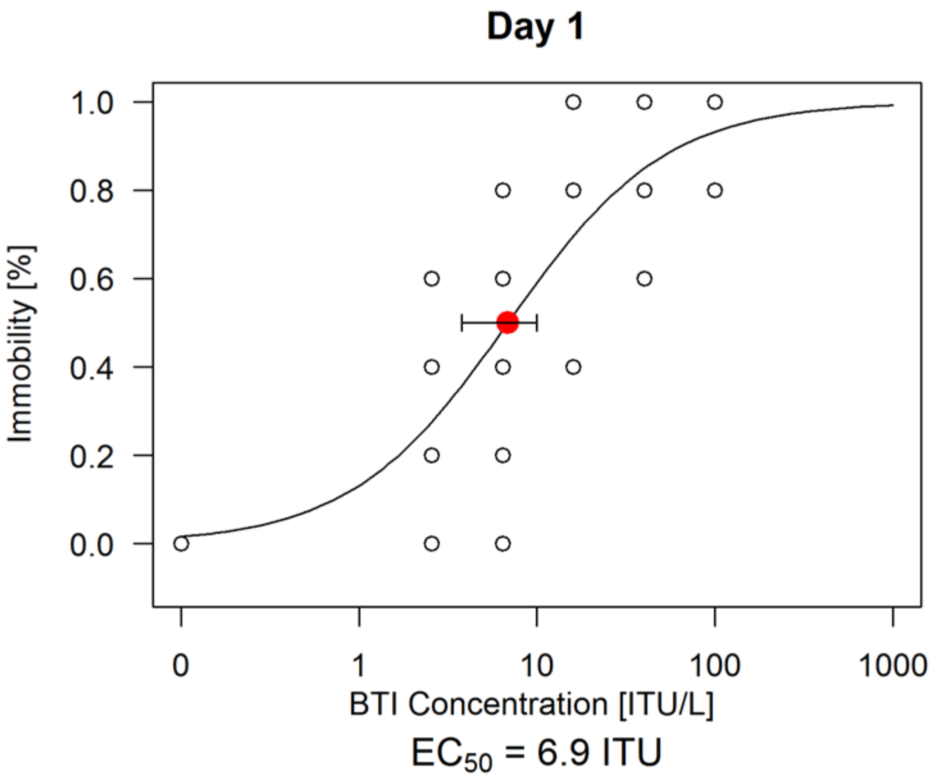

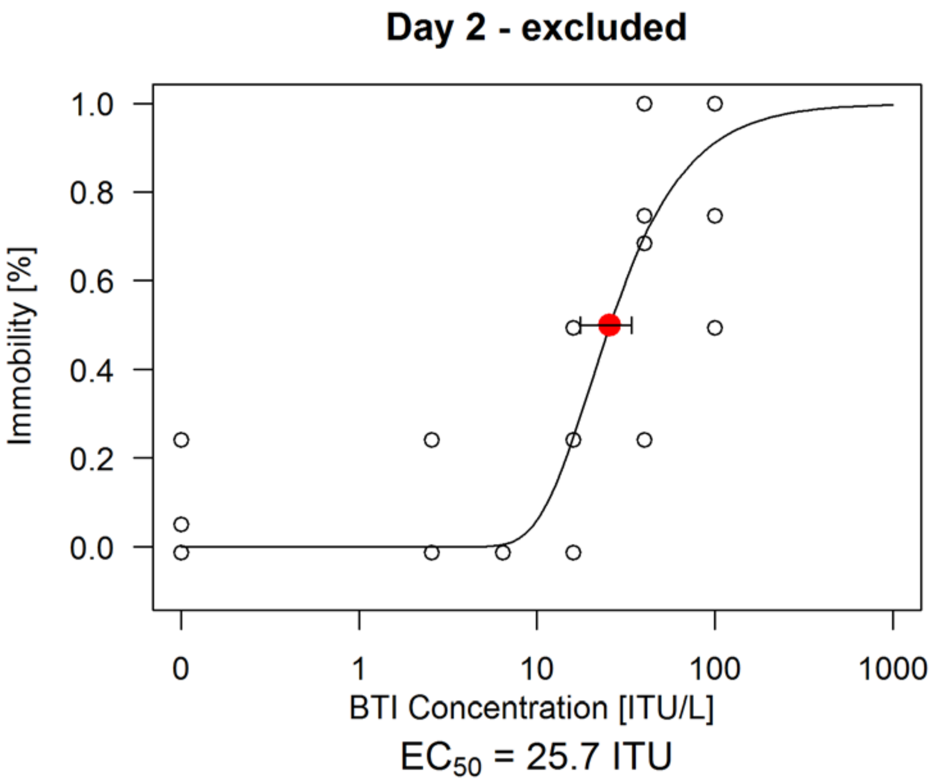
Day 2 - excluded
1.0
0.8
0.6
0.4
0.2
0.0
Immobility [%]
0
1
10
100
1000
BTI Concentration [ITU/L]
EC50 = 25.7 ITU

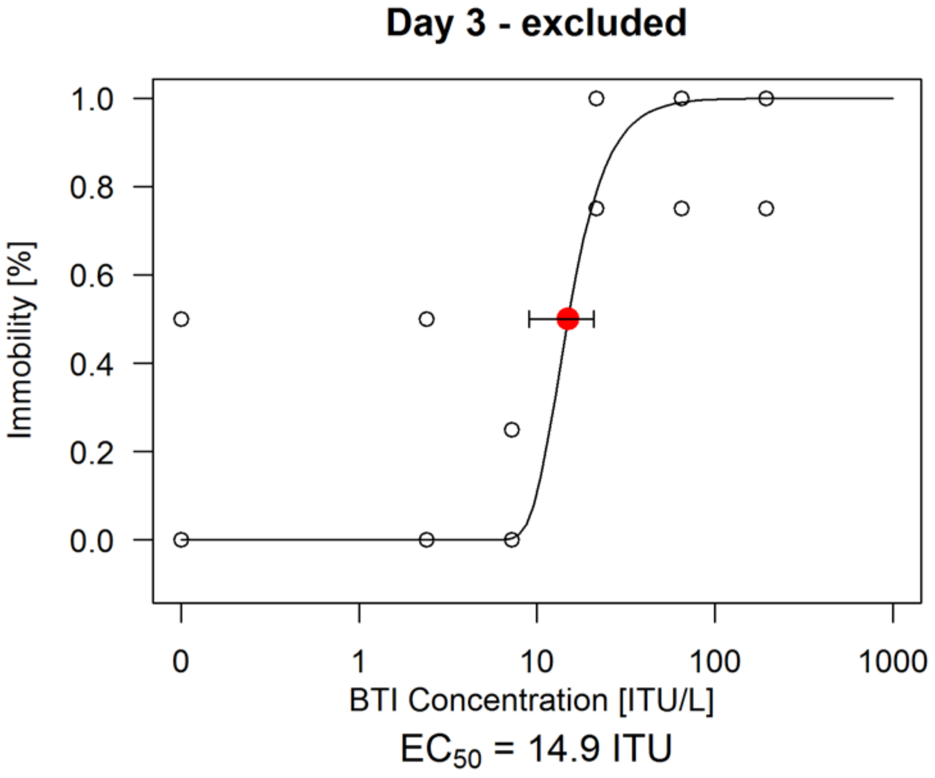
Day 3 - excluded
1.0
0.8
0.6
0.4
0.2
0.0
Immobility [%]
0
1
10
100
1000
BTI Concentration [ITU/L]
EC50 = 14.9 ITU

Day 4

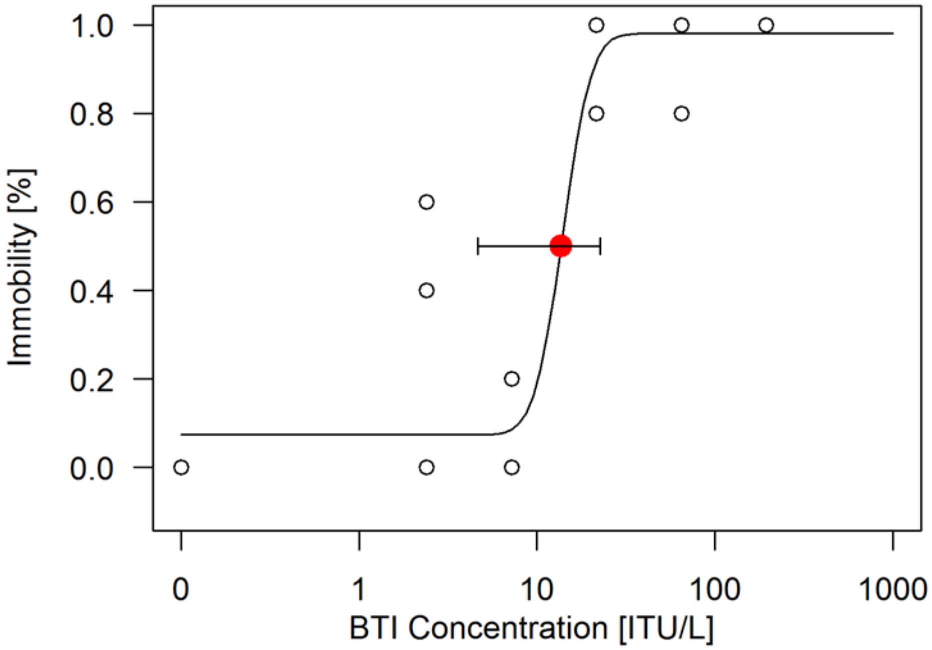

EC_{50} = 13.6 ITU

Day 5

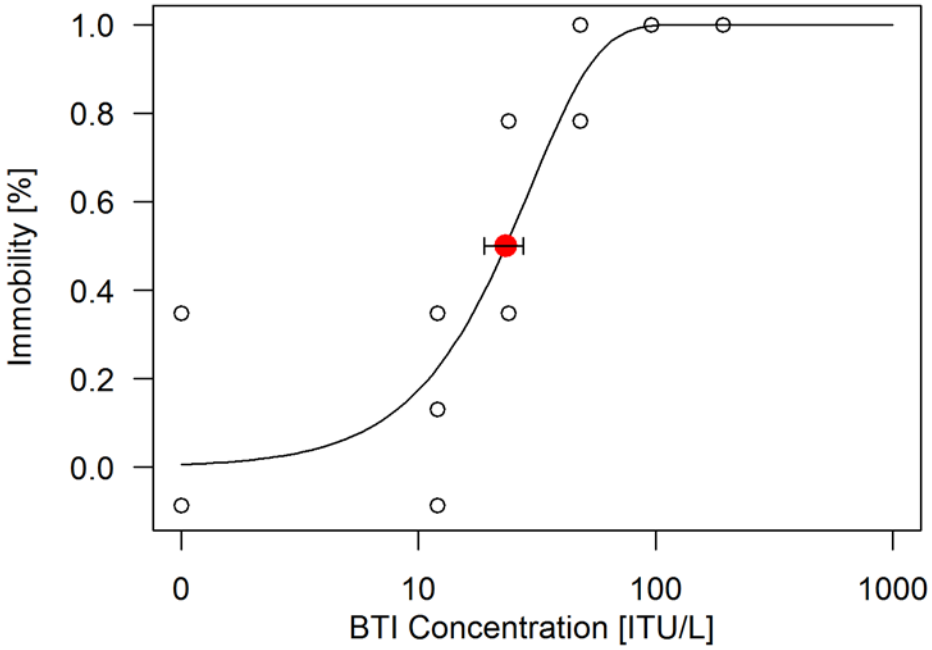

EC_{50} = 23.3 ITU

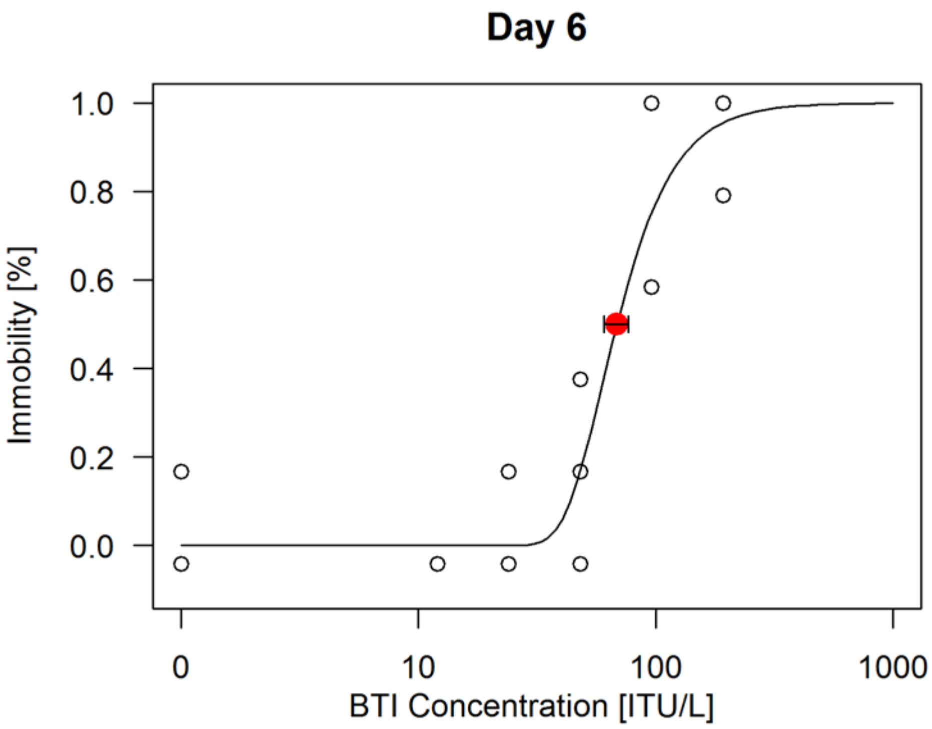

EC_{50} = 68.7 ITU

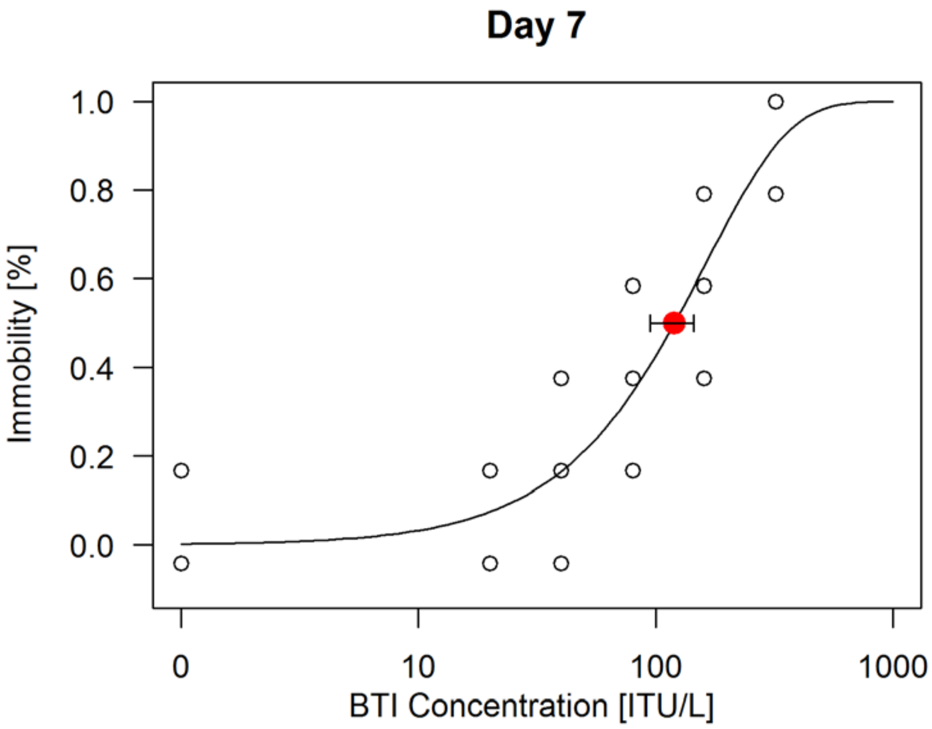

EC_{50} = 120.0 ITU

Day 8

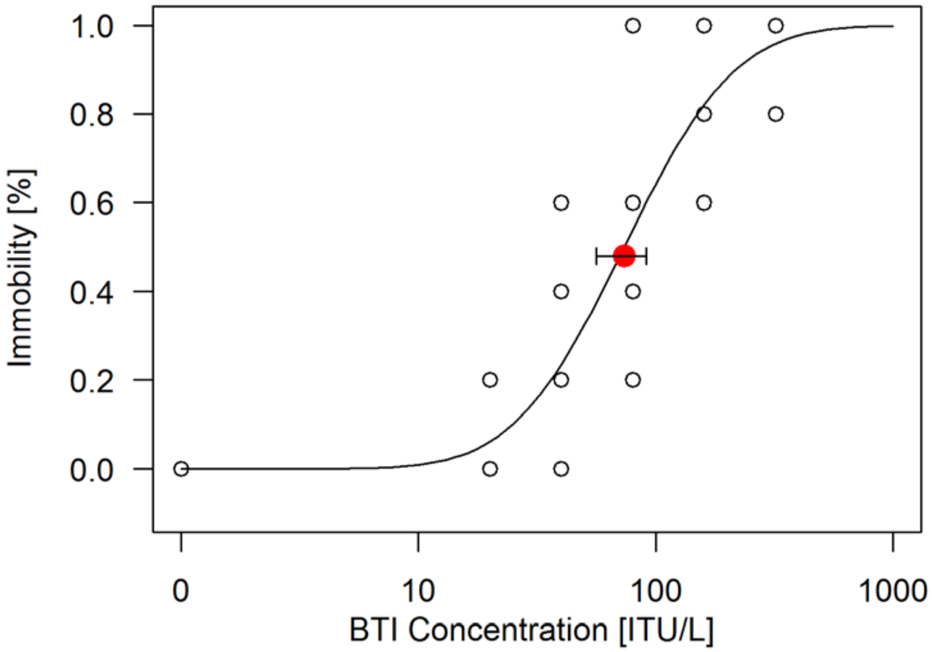

EC_{50} = 73.7 ITU

Day 9

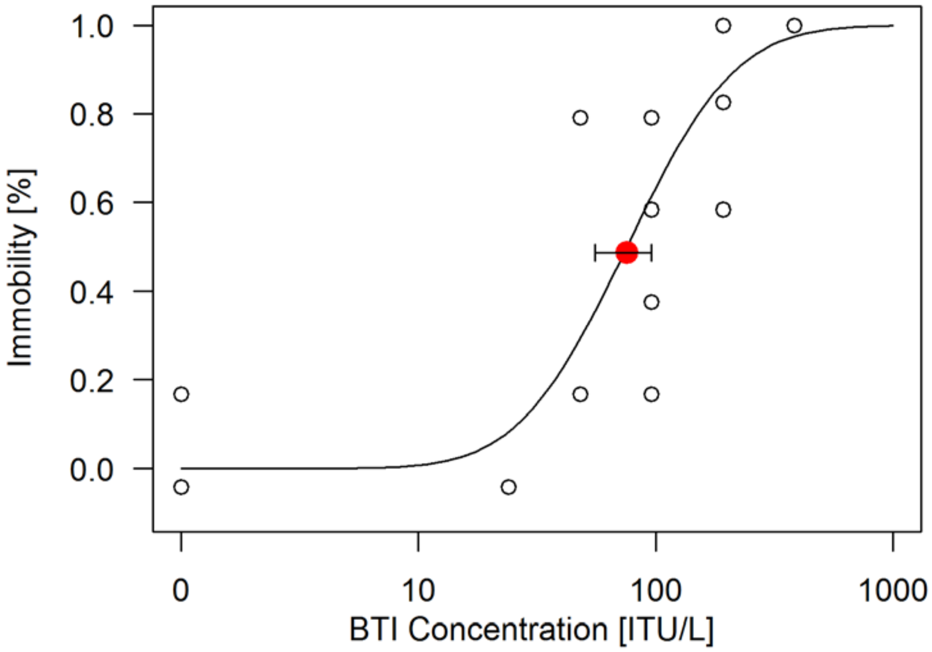

EC_{50} = 75.6 ITU

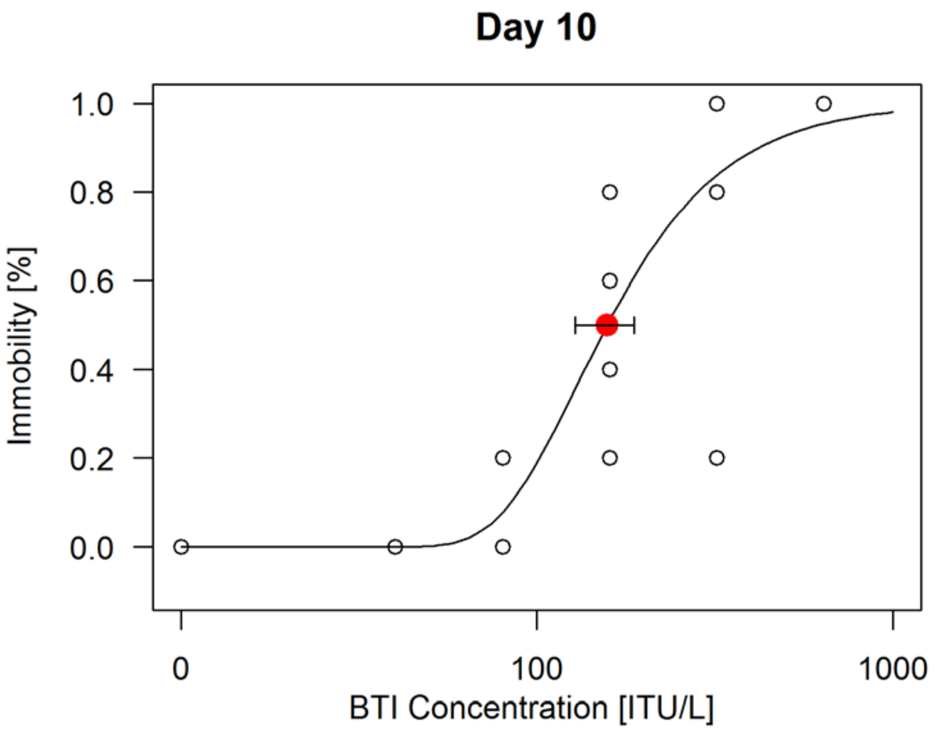

EC_{50} = 157.5 ITU

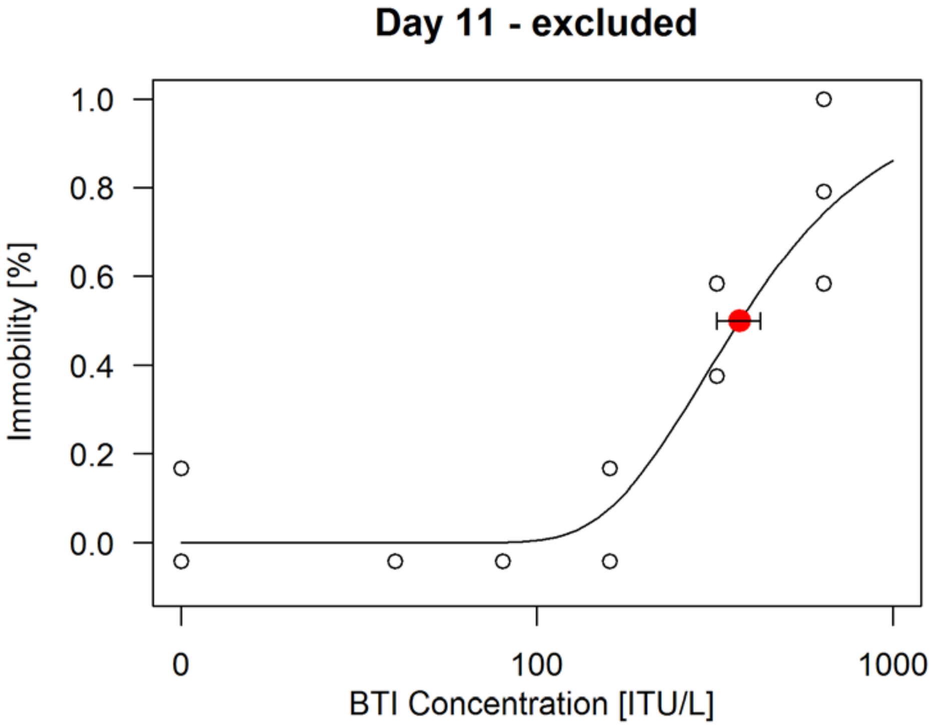

EC_{50} = 371.3 ITU

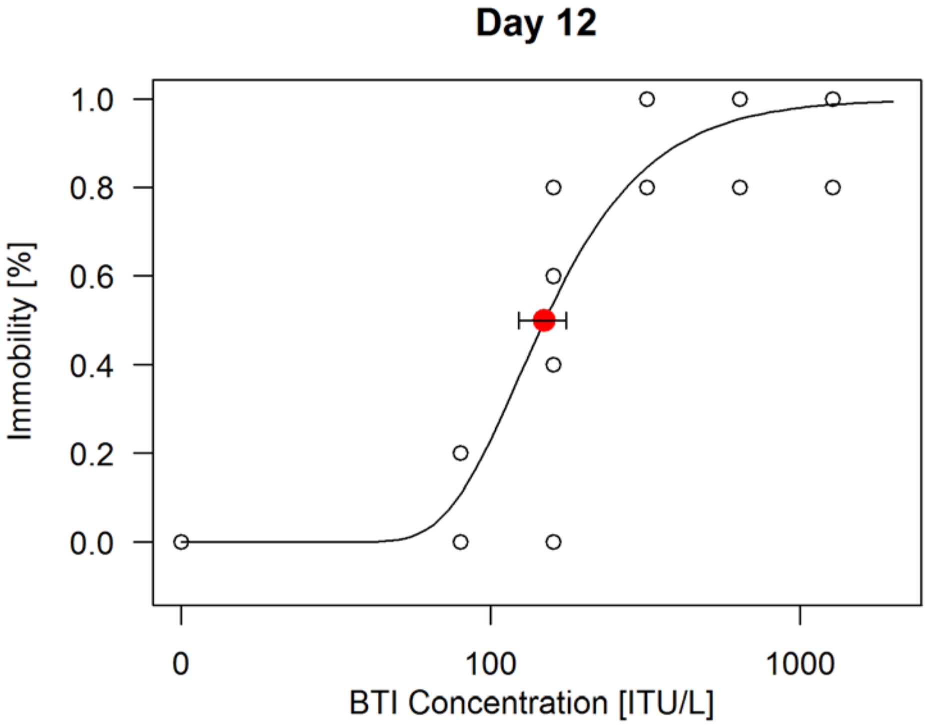

EC_{50} = 149.7 ITU

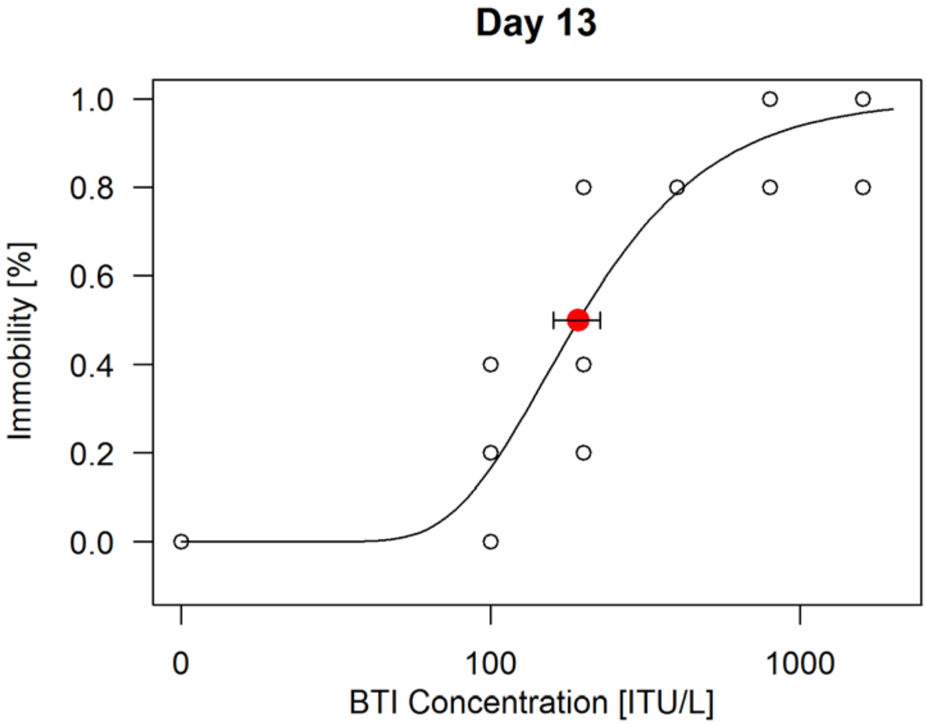

EC_{50} = 192.6 ITU

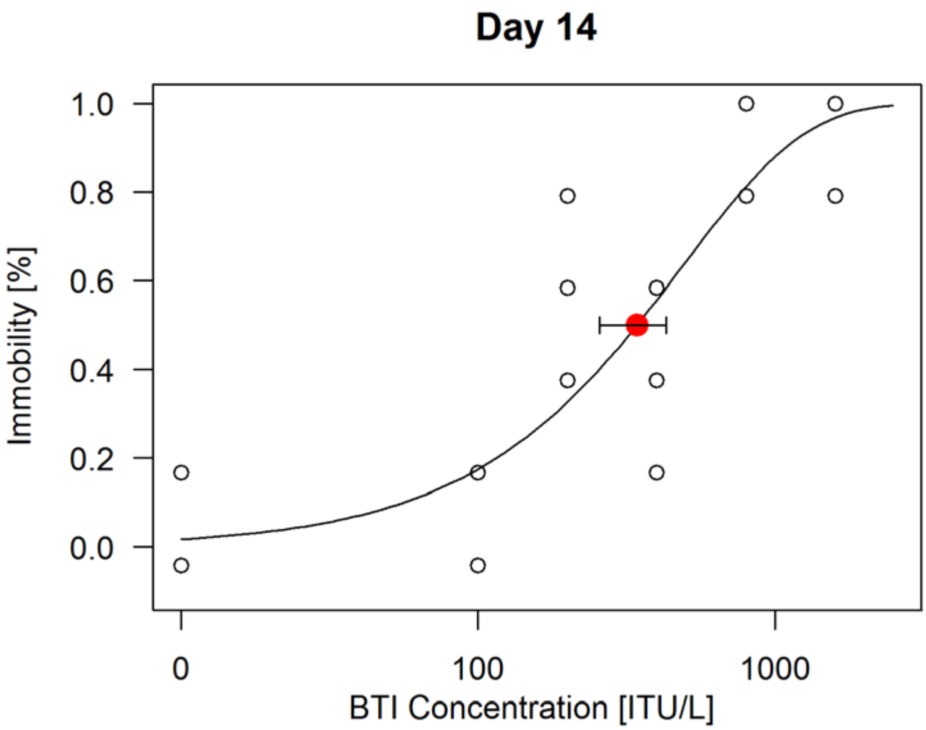

EC_{50} = 343.3 ITU

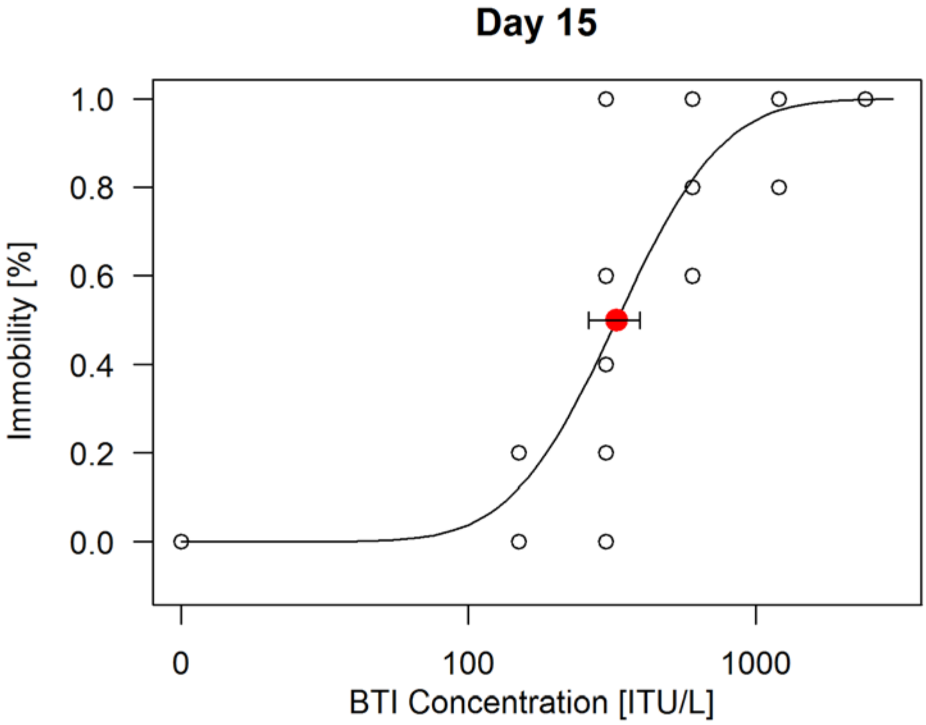

EC_{50} = 328.0 ITU

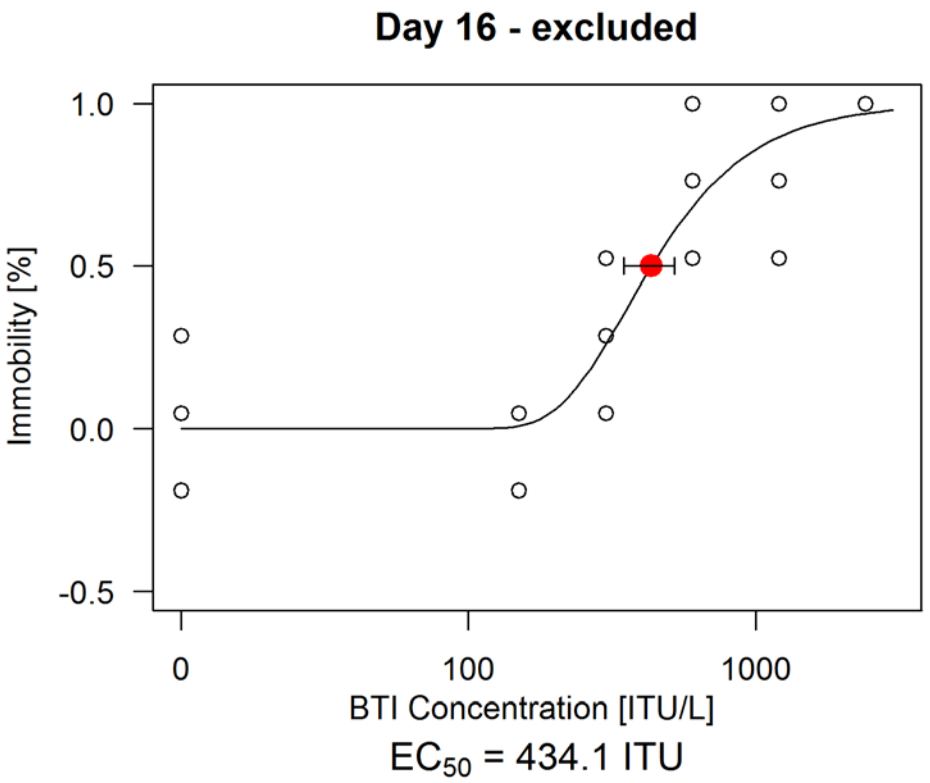
Day 16 - excluded
1.0
0.5
0.0
-0.5
Immobility [%]
0
100
1000
BTI Concentration [ITU/L]
EC50 = 434.1 ITU

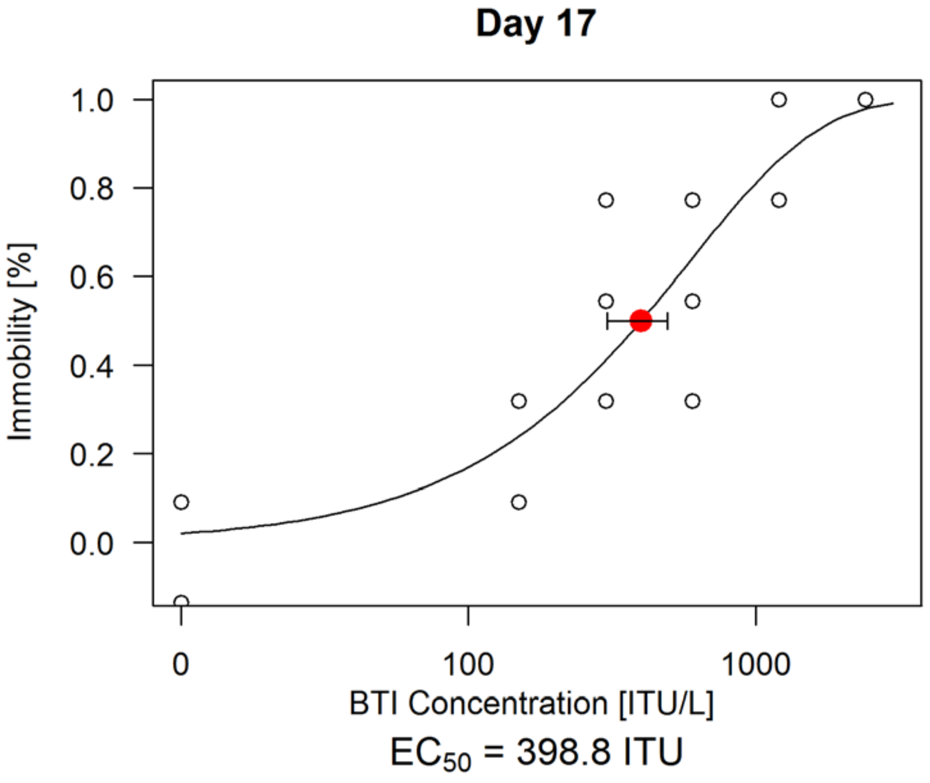
Day 17
1.0
0.8
0.6
0.4
0.2
0.0
Immobility [%]
0
100
1000
BTI Concentration [ITU/L]
EC50 = 398.8 ITU

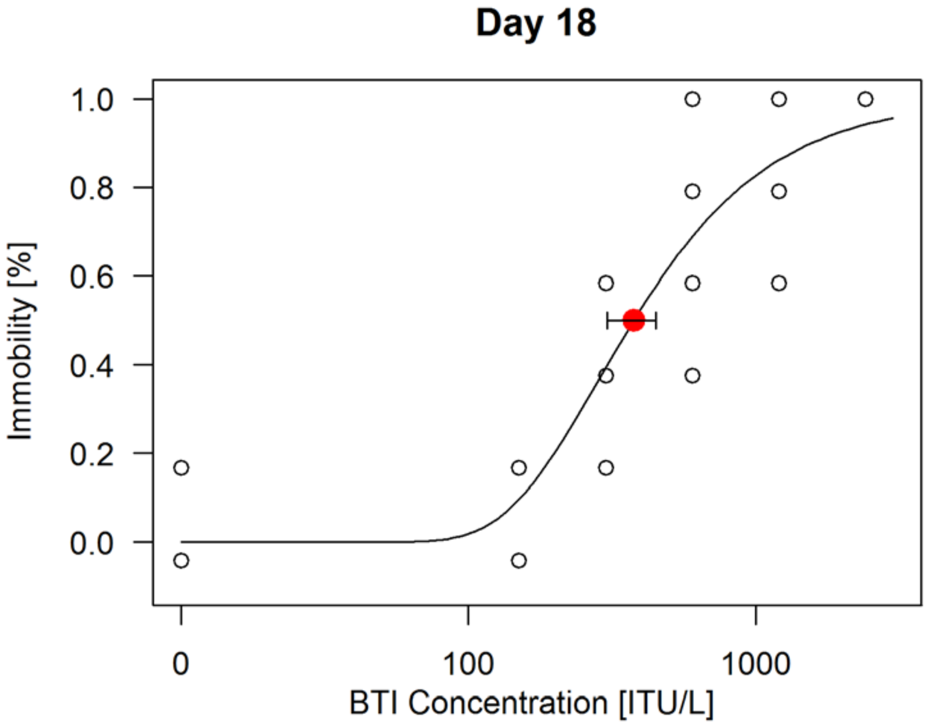

EC_{50} = 376.1 ITU

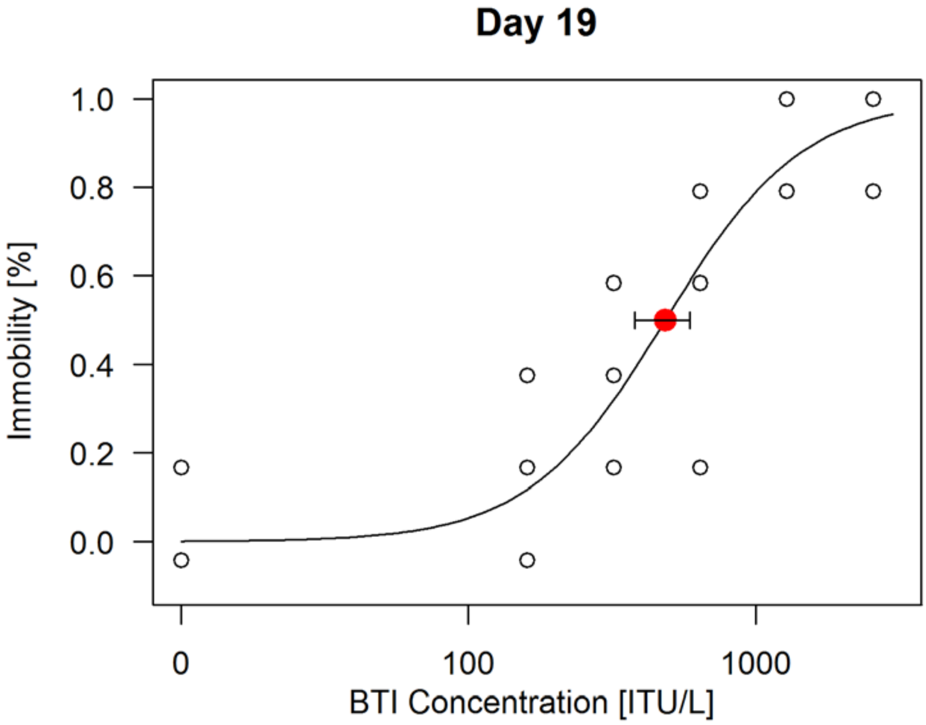

EC_{50} = 483.8 ITU

Day 20

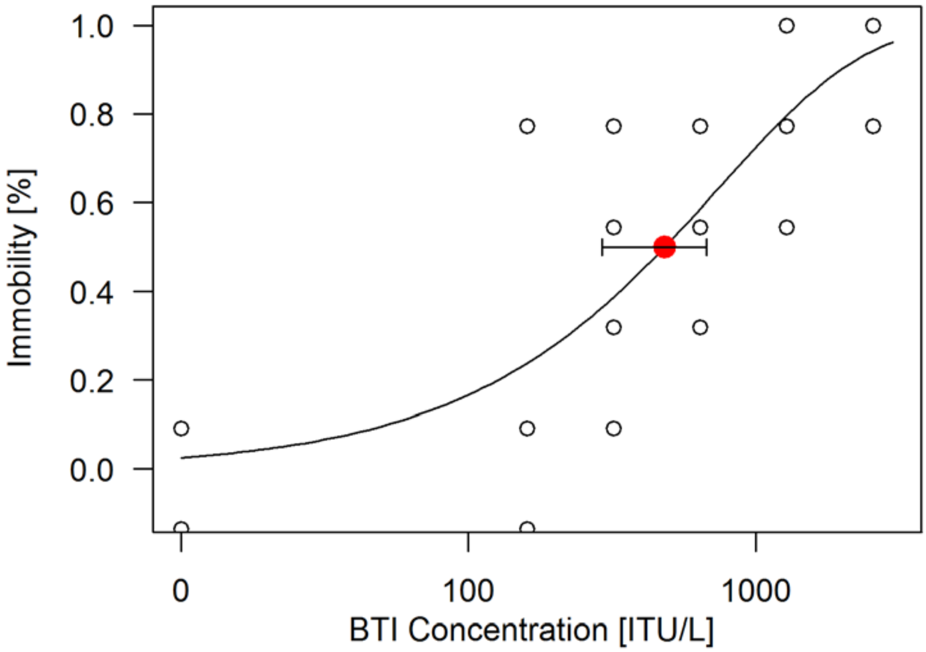

EC_{50} = 482.2 ITU

Day 21

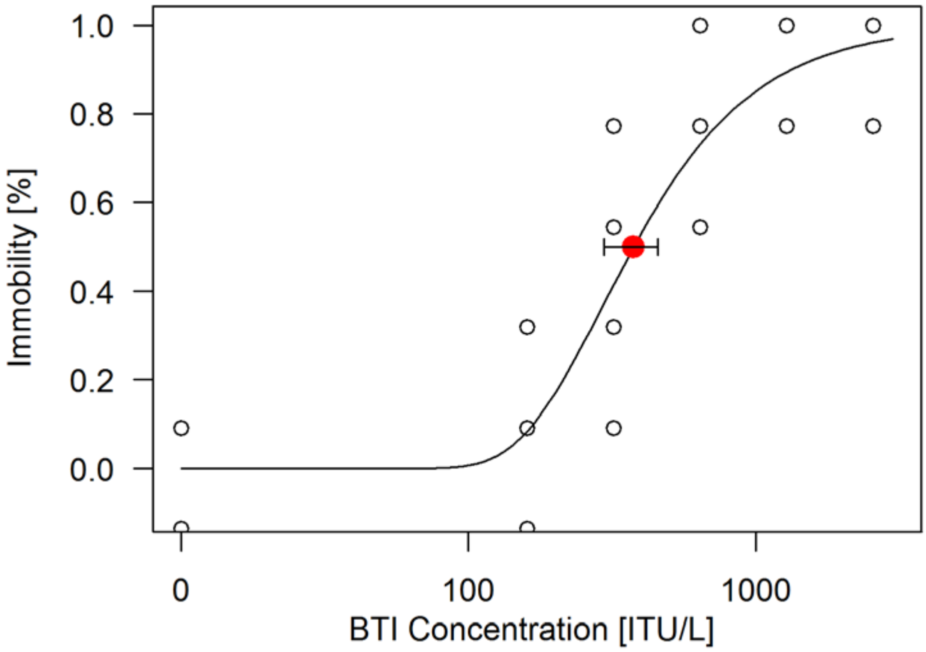

EC_{50} = 376.0 ITU

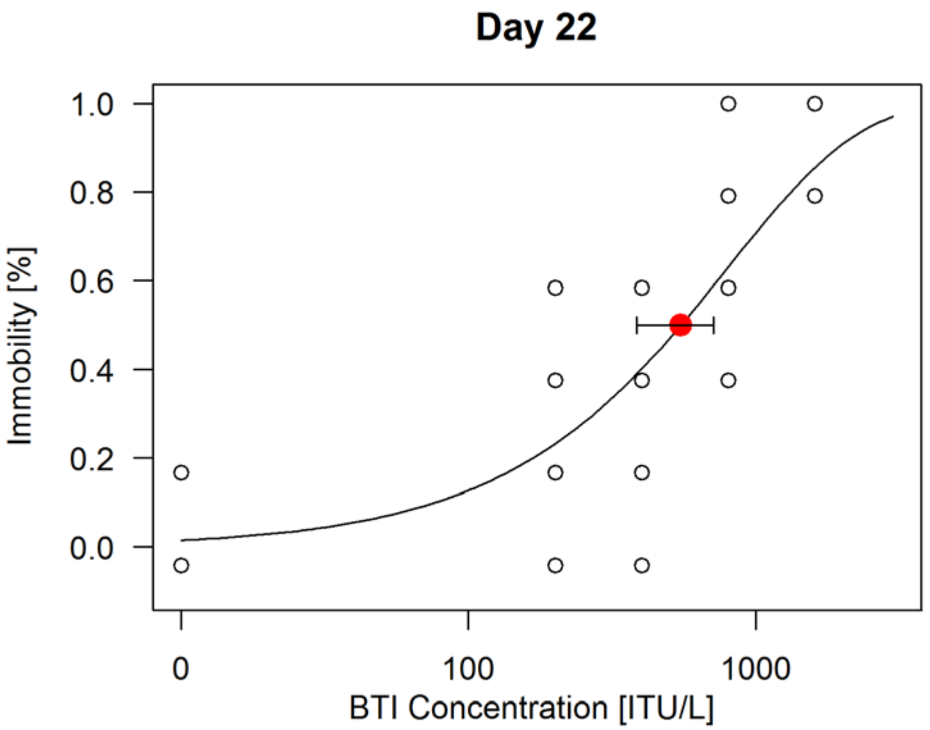

EC_{50} = 549.1 ITU

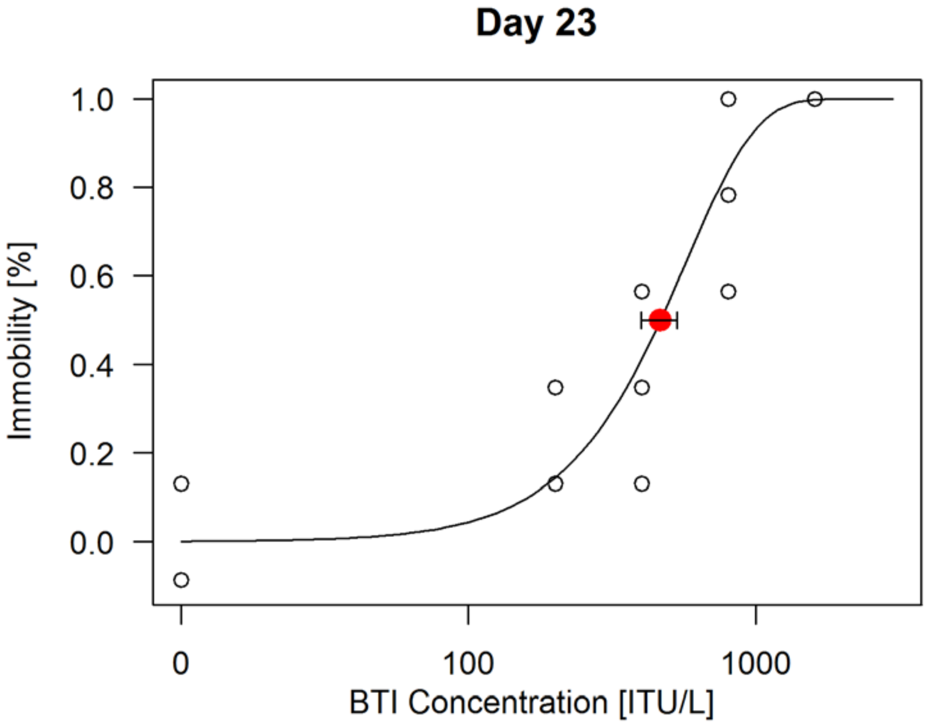

EC_{50} = 465.8 ITU

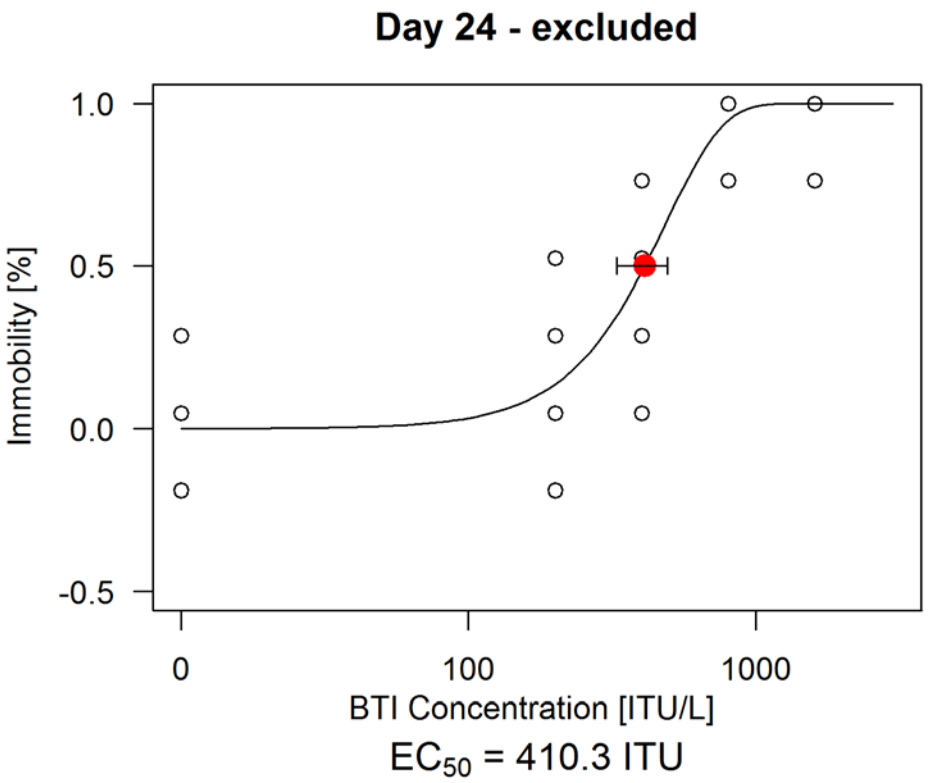
Day 24 - excluded
1.0
0.5
0.0
-0.5
Immobility [%]
0
100
1000
BTI Concentration [ITU/L]
EC50 = 410.3 ITU

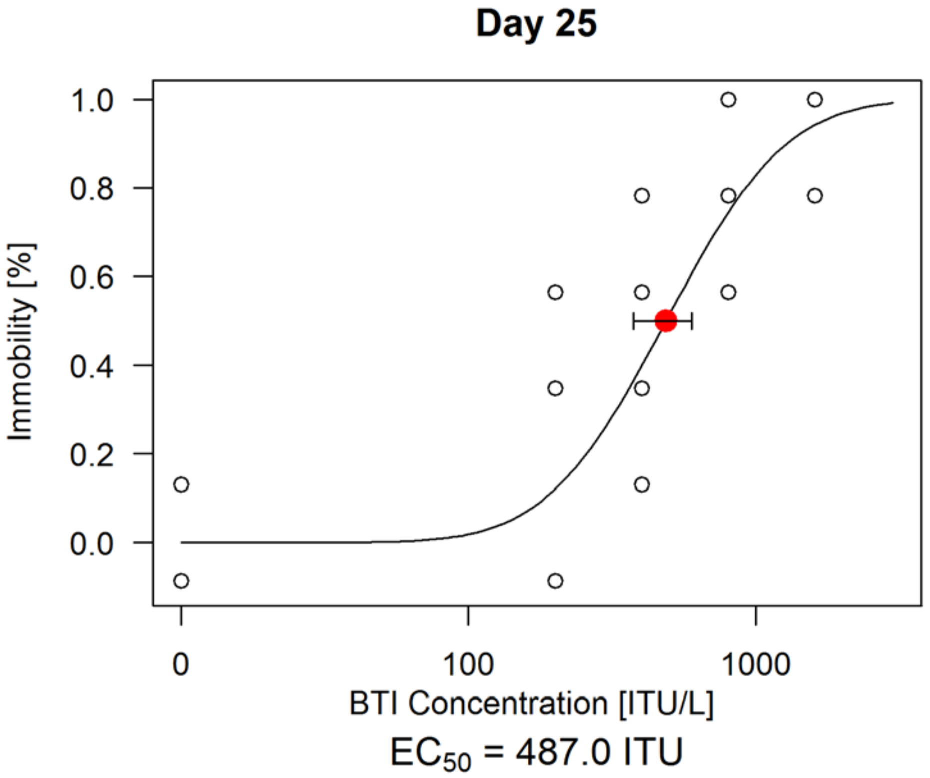
Day 25
1.0
0.8
0.6
0.4
0.2
0.0
Immobility [%]
0
100
1000
BTI Concentration [ITU/L]
EC50 = 487.0 ITU

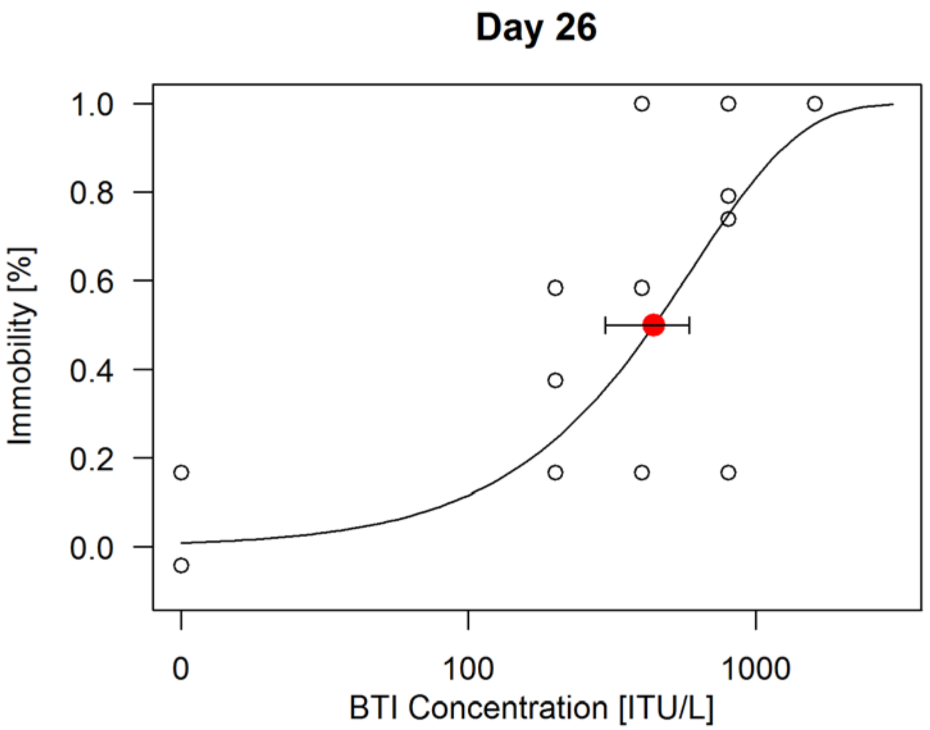

EC_{50} = 442.6 ITU

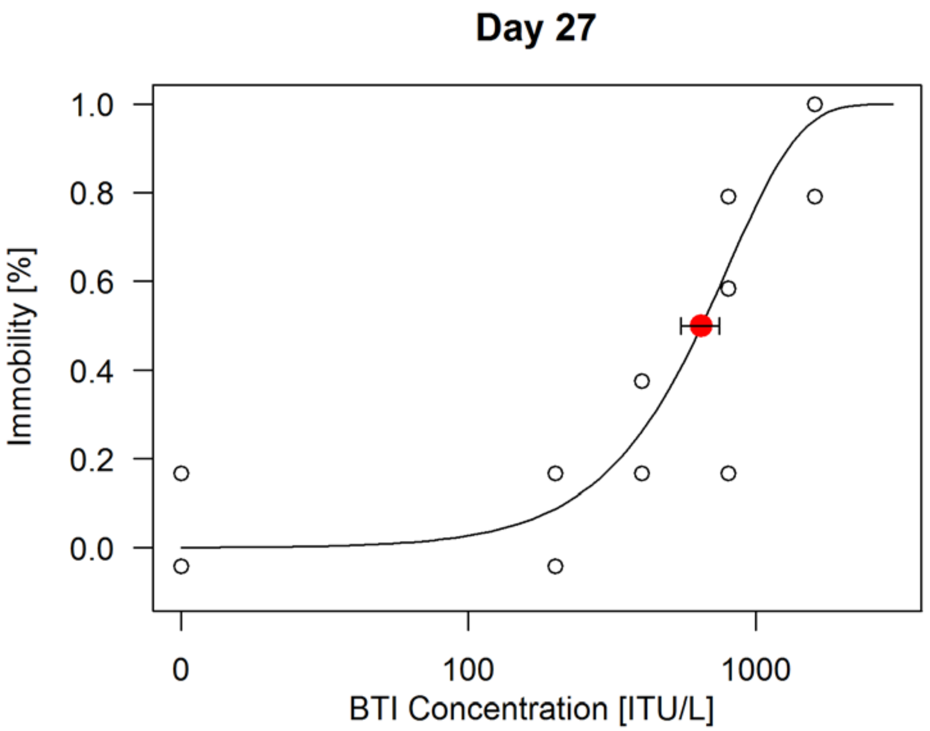

EC_{50} = 647.2 ITU

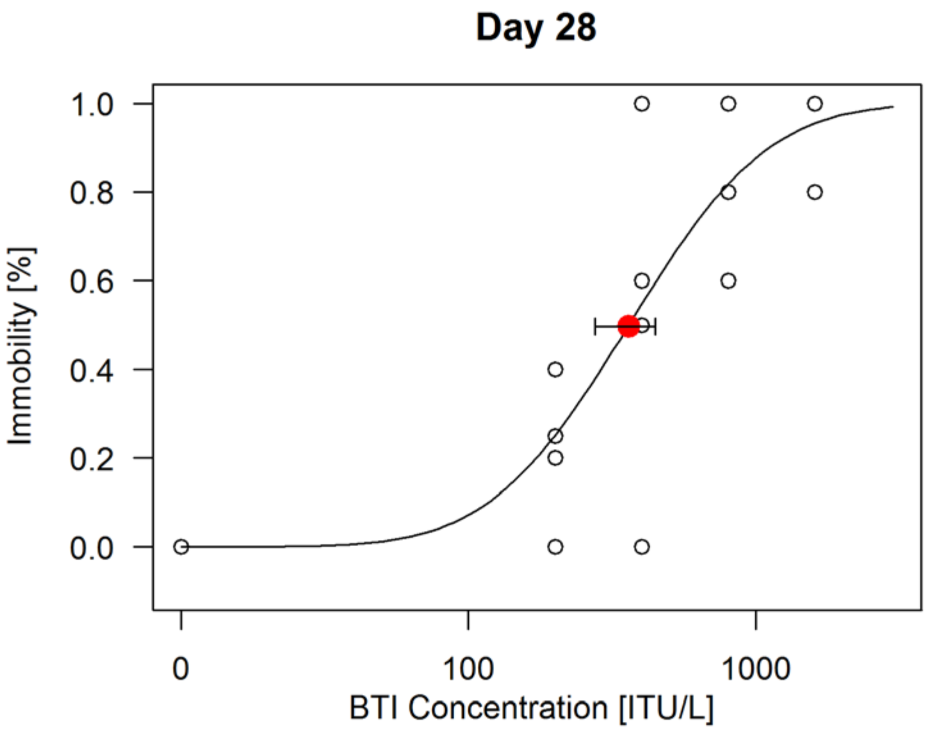

EC_{50} = 361.1 ITU

Figure S6: Summary of the literature review of EC50 values. The positions of the EC50 values were jittered to improve readability. Blue colour represents species with records in Europe, red represents non-European species. Different species are presented with different symbols. For further analysis only EC50 values dedicated to a certain larval stage were used.

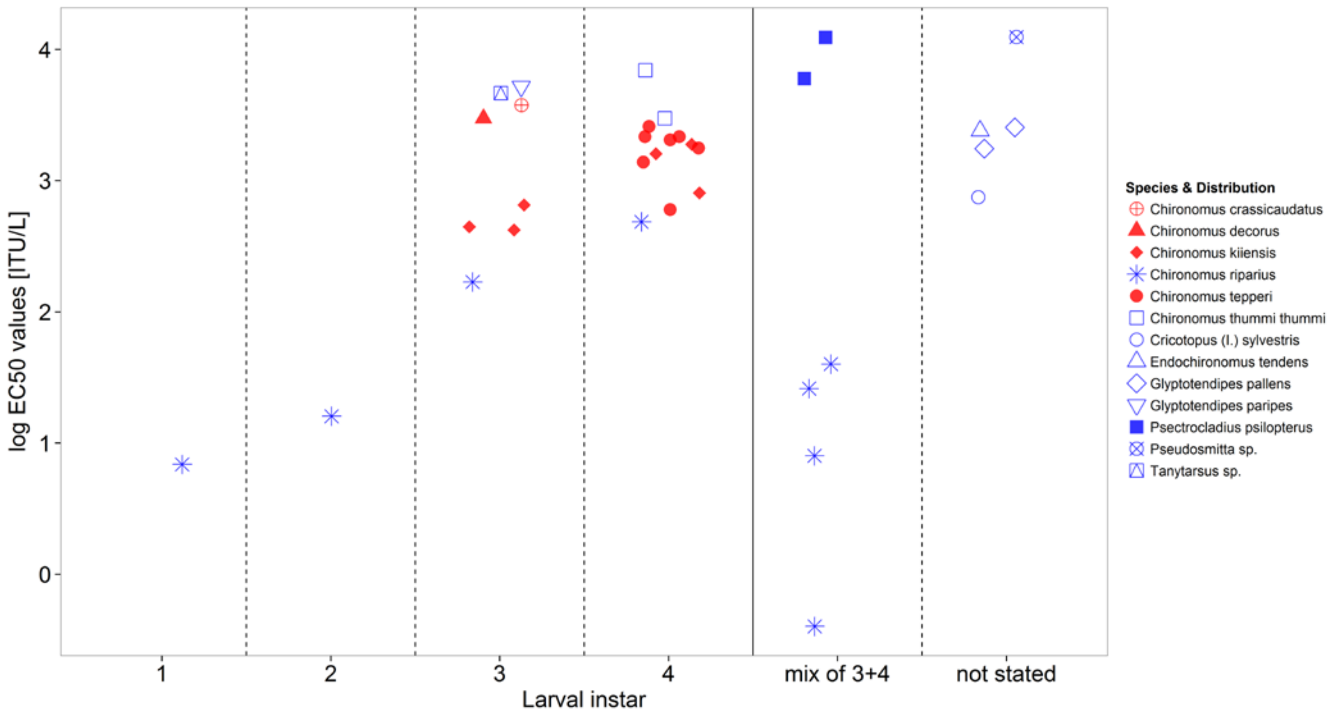

Table S7: Information and workflow of the statistical analysis of influential parameters on EC50 values based on Figure S6:

A literature research of laboratory values for chironomids and Bti was performed. Only studies with a stated ITU/mg content for the tested Bti product were included [5–7,13,21,26–28]. Different test parameters are known to influence acute toxicity studies such as sediment presence or absence [6,27], test duration [19,29], larval stage [20,22,28–30], test organism density per replicate [6,27] and test species [2,7,26]. Those factors were included as explanatory variables in a linear model. After logarithmic transformation of the obtained EC50 values to normalize data, linear models were applied to identify factors with highest explanatory power for EC50 values. Only EC50 values dedicated to a certain larval stage were used. Different linear models were tested with ANOVA to get the most parsimonious model.

1) We checked for covariate (visually checked via scatterplot, no covariate exist) and included all potentially influencing factors like species, larval instar, test duration (time), product, density of the larvae and substrate in the first model.

```
> summary(firstlm)

Call:
lm(formula = logEC50 ~ species + instar + time + product + density + 
    substrate, data = lvgl)

Residuals:
      Min        1Q    Median        3Q       Max 
-0.277183 -0.106189  0.009764  0.106189  0.192639 

Coefficients: (5 not defined because of singularities)
                            Estimate Std. Error t value Pr(>|t|)    
(Intercept)                  0.84868    0.32507   2.611   0.0311 *  
speciesChironomus riparius  -0.36264    0.31486  -1.152   0.2827    
speciesChironomus tepperi    0.04661    0.26110   0.178   0.8628    
speciesChironomus thummi thummi  0.39372    0.21452   1.835   0.1038    
instar                       0.60359    0.08257   7.310 8.31e-05 ***
time                              NA         NA      NA       NA    
productBactimos powder BRB 0032   NA         NA      NA       NA    
productoil miscible supension -0.19698    0.21051  -0.936   0.3768    
producttechnical material    0.04728    0.21051   0.225   0.8279    
productTeknar SC             0.10199    0.29770   0.343   0.7407    
productVectoBac WDG         -0.25430    0.24307  -1.046   0.3261    
productwettable powder            NA         NA      NA       NA    
density                           NA         NA      NA       NA    
substratesand                     NA         NA      NA       NA    
---
Signif. codes:  0 '***' 0.001 '**' 0.01 '*' 0.05 '.' 0.1 ' ' 1

Residual standard error: 0.2105 on 8 degrees of freedom
Multiple R-squared:  0.9633,	Adjusted R-squared:  0.9265 
F-statistic: 26.22 on 8 and 8 DF,  p-value: 5.83e-05
```

2) Variables with still missing values were kicked out of the analysis. Instar was highly significant in the first model building step. Therefore, the second model consisted of the following parameters: species, larval instar and product, not significant parameters as test duration (time), density and substrate.

```
> second <-lm(logEC50~ species + instar + product, data=lvgl)
> summary(second)

Call:
lm(formula = logEC50 ~ species + instar + product, data = lvgl)

Residuals:
     Min       1Q   Median       3Q      Max 
-0.27718 -0.01206  0.00000  0.08454  0.19264 

Coefficients: (3 not defined because of singularities)
                                Estimate Std. Error t value Pr(>|t|)    
(Intercept)                      1.76328    0.32507   5.424 0.000628 ***
speciesChironomus decorus       -0.09982    0.29770  -0.335 0.746033    
speciesChironomus kiiensis      -0.91459    0.26110  -3.503 0.008046 ** 
speciesChironomus riparius      -1.27723    0.34085  -3.747 0.005647 ** 
speciesChironomus tepperi       -0.86799    0.30894  -2.810 0.022858 *  
speciesChironomus thummi thummi -0.52087    0.27072  -1.924 0.090548 .  
speciesGlyptotendipes paripes    0.14281    0.29770   0.480 0.644284    
speciesTanytarsus sp.            0.09155    0.29770   0.308 0.766300    
instar                           0.60359    0.08257   7.310 8.31e-05 ***
productBactimos powder BRB 0032       NA         NA      NA       NA    
productIPS-78 (WP)                    NA         NA      NA       NA    
productoil miscible supension   -0.19698    0.21051  -0.936 0.376791    
producttechnical material        0.04728    0.21051   0.225 0.827904    
productTeknar SC                 0.10199    0.29770   0.343 0.740735    
productVectoBac WDG             -0.25430    0.24307  -1.046 0.326059    
productwettable powder                NA         NA      NA       NA    
---
Signif. codes:  0 '***' 0.001 '**' 0.01 '*' 0.05 '.' 0.1 ' ' 1

Residual standard error: 0.2105 on 8 degrees of freedom
Multiple R-squared:  0.9699,	Adjusted R-squared:  0.9248 
F-statistic:  21.5 on 12 and 8 DF,  p-value: 9.131e-05
```

3) Species and instar are significant; product is not significant and could be left out in the next model approach:

```
> third <-lm(logEC50 ~ species + instar, data=lvgl)
> summary(third)

Call:
lm(formula = logEC50 ~ species + instar, data = lvgl)

Residuals:
     Min       1Q   Median       3Q      Max 
-0.39930 -0.00901  0.00976  0.07052  0.23417 

Coefficients:
                               Estimate Std. Error t value Pr(>|t|)    
(Intercept)                     1.76328    0.32718   5.389 0.000163 ***
speciesChironomus decorus      -0.09982    0.29963  -0.333 0.744790    
speciesChironomus kiiensis     -0.96449    0.23259  -4.147 0.001354 ** 
speciesChironomus riparius     -1.53153    0.24050  -6.368 3.57e-05 ***
speciesChironomus tepperi      -1.00017    0.24653  -4.057 0.001590 ** 
speciesChironomus thummi thummi -0.52087    0.27247  -1.912 0.080093 .  
speciesGlyptotendipes paripes   0.14281    0.29963   0.477 0.642206    
speciesTanytarsus sp.           0.09155    0.29963   0.306 0.765191    
instar                          0.60359    0.08310   7.263 9.97e-06 ***
---
Signif. codes:  0 '***' 0.001 '**' 0.01 '*' 0.05 '.' 0.1 ' ' 1

Residual standard error: 0.2119 on 12 degrees of freedom
Multiple R-squared:  0.9543,	Adjusted R-squared:  0.9238 
F-statistic: 31.32 on 8 and 12 DF,  p-value: 6.791e-07
```

4) Model parameters were checked visually. To ensure the most parsimonious model a fourth model with instar only was build:

```
Call:
lm(formula = logEC50 ~ instar, data = lvgl)

Residuals:
     Min       1Q   Median       3Q      Max 
-0.82004 -0.46752 -0.07522  0.11445  1.01837 

Coefficients:
            Estimate Std. Error t value Pr(>|t|)    
(Intercept)   0.6756     0.5373   1.257  0.22389    
instar        0.6743     0.1548   4.356  0.00034 ***
---
Signif. codes:  0 '***' 0.001 '**' 0.01 '*' 0.05 '.' 0.1 ' ' 1

Residual standard error: 0.5571 on 19 degrees of freedom
Multiple R-squared:  0.4997,	Adjusted R-squared:  0.4733 
F-statistic: 18.97 on 1 and 19 DF,  p-value: 0.0003402
```

5) The adjusted R^2 of the fourth model is 0.47 which is about half of the adjusted R^2 of the third model (0.92). The third and the fourth model were statistically significant different, which was tested by an ANOVA. Due to the better explanatory power of the data, the third model was chosen as the best model to describe the dispersion of the EC50 data. Instar and species were revealed as most influencing factors.

Figure S7: Head capsule width (HCW) is plotted versus head capsule length (HCL) of *C. riparius* larvae. The larvae were assigned to four clusters representing the four larval instars.

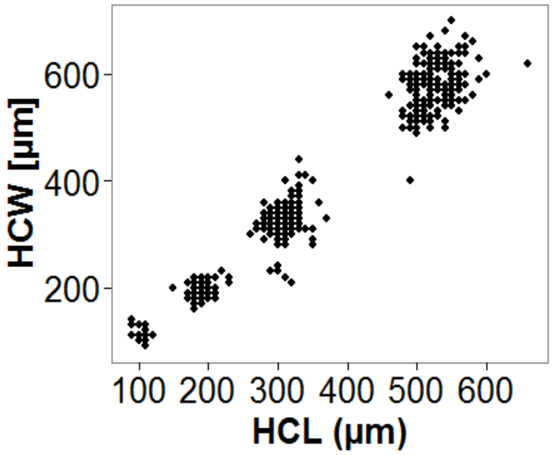

Table S8: Mean values for length, head capsule width (HCW) and head capsule length (HCL) in the different larval instars were calculated with randomly chosen *C. riparius* larvae out of the culture vessel at the respective test days.

Larval stage	Length (mm)	HCW (µm)	HCL (µm)
1	1.6	104	115
2	3.3	192	200
3	6.7	308	332
4	10.8	525	582

Figure S9: Calculation of the mean 48 h EC50 values of the four different larval stages. All replicates of the tests are included in the figures below (transformed with Abbotts formula to adjust for control mortality).

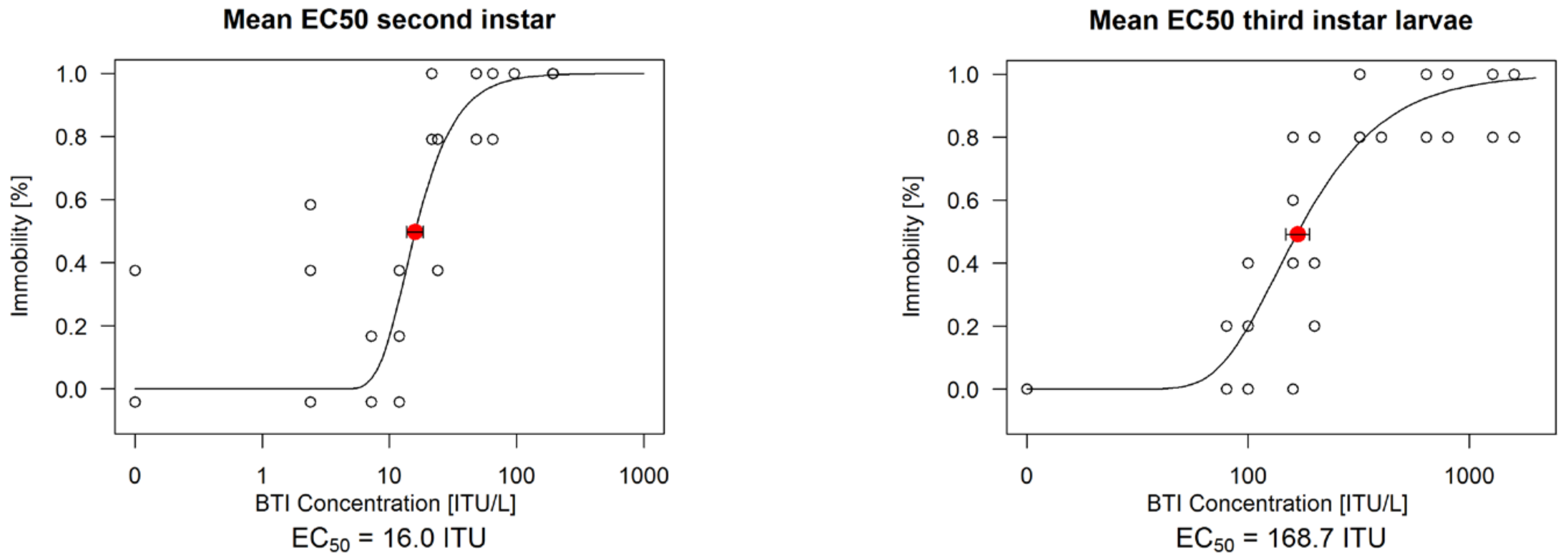

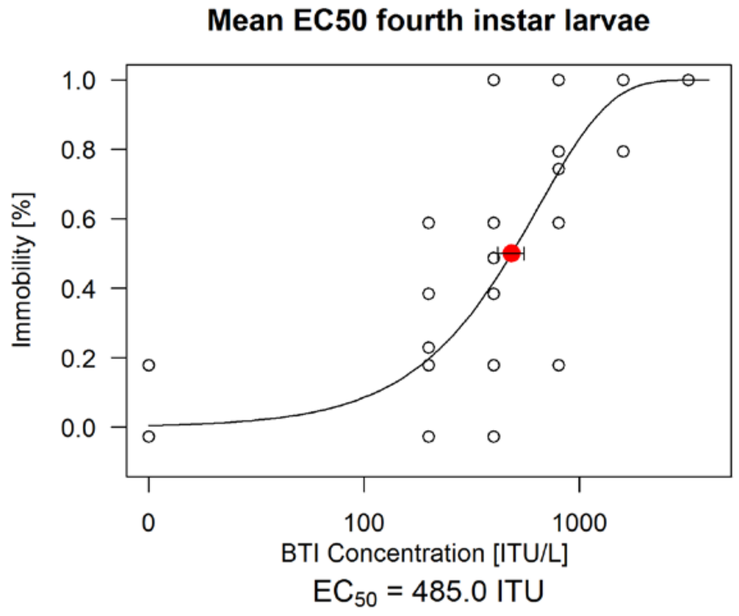

Table S10: Detailed information on the EC50 calculations such as the 95% Confidence Interval (CI), the chosen model, Akaikes information criterion and the lack of fit that were calculated with the package “drc” in R.

Larval stage	Test day	EC50	lower CI	upper CI	Model		Akaikes Information Criterion	Lack of fit
1	Day 2	6.87	3.77	9.98	log-logistic dose-response model with two parameters	LL.2	-10.14	0.80
2	Day 4+5	16.01	13.60	18.42	two-parameter Weibull function	W1.2	-34.07	0.00
3	Day 12+13	168.66	147.91	189.41	two-parameter Weibull function	W1.2	-55.12	0.69
4	Day 26,27,28	484.96	416.60	553.33	two-parameter Weibull function	W2.2	-48.93	1.00

Table S10: Mean EC50 values were analysed for statistically significant differences among the four larval instars using confidence interval overlap testing.

	Day	Mean EC50 (ITU/L)	Mean CI (ITU/L)	standard error (ITU/L)	95% Confidential interval (ITU/L)	p-value	increase
First instar	1	6.9		1.5	3.8 - 10.0		
		to second instar	-9.134618	1.934067	-9.2 - -9.1	0.002758821	2.3
		to third instar	-161.7841	10.47816	-161.9 - -161.7	5.46E-39	24.4
		to fourth instar	-478.0903	34.43345	-478.4 - 477.7	3.12E-31	70.3
Second instar	4, 5	16		1.2	13.6 - 18.4		
		to third instar	-152.6495	10.43795	-152.8 - -152.5	8.20E-35	10.5
		to fourth instar	-468.9556	34.42124	-469.3 - -468.6	5.10E-30	30.3
Third instar	12, 13	168.7		10.4	147.9 - 189.4		
		to fourth instar	-316.3062	35.92872	-316.7 - -315.9	5.48E-12	2.8
Fourth instar	26-28	485		34.4	416.6 - 553.3		
